# U.S. policy on wireless technologies and public health protection: regulatory gaps and proposed reforms

**DOI:** 10.3389/fpubh.2025.1677583

**Published:** 2025-12-19

**Authors:** Theodora Scarato

**Affiliations:** Wireless and EMF Program, Environmental Health Sciences, Bozeman, MT, United States

**Keywords:** radio-frequency, electromagnetic fields, Federal Communications Commission, ICNIRP, wireless technology, non-ionizing, cell tower, cell phone

## Abstract

The current U.S. regulatory framework governing non-ionizing radiofrequency radiation (RFR) used in all wireless technology is outdated and lacks adequate protection, oversight, and enforcement. The U.S. Federal Communications Commission (FCC) was given regulatory jurisdiction by the U.S. Congress in 1996 over RFR exposure standards setting even though FCC has no in-house expertise regarding health or environmental effects from RFR. FCC is a licensing/engineering entity that relies on other government agencies for guidance on ambient exposures and devices. However, all relevant civilian public health and environmental agencies have been defunded from non-ionizing radiation research activities and oversight. Thus, current regulations have remained unchanged since 1996. Human exposure limits are designed to protect against short-term high-intensity effects, not today's long-term chronic low-intensity exposures. Scientific evidence indicates that children's thinner skulls, unique physiology, and more conductive tissues result in significantly higher RFR absorption rates deeper into critical brain regions, which are still in development and thus more sensitive to environmental insults. However, current policies offer no safeguards for children/pregnancy or vulnerable populations. Growing research also indicates risks to wildlife, especially pollinators. In 2021, a U.S. federal court mandated that the FCC show proper review of growing scientific evidence, after a cursory FCC re-approval of limits in 2019, but FCC has yet to respond. This paper explores regulatory infrastructure deficiencies, including the absence of monitoring/oversight, premarket safety testing, post-market surveillance, emissions compliance/enforcement, occupational safety, and wildlife protection. Compliance tests for cell phones do not reflect real-world consumer use and can therefore camouflage exposures that exceed even FCC's outdated limits. Other countries enforce stricter limits, robust monitoring, transparency measures, and compliance programs with additional policies to protect children. Also discussed is the chronic revolving door between FCC leadership and the wireless industry, resulting in a state of regulatory capture. Policy recommendations for common-sense reforms are made for reinvigorating independent research, developing science-based safety limits, ensuring pre- and post-market surveillance, and improving oversight/enforcement, as well as implementing risk mitigation to reduce exposures to children, vulnerable groups, and wildlife.

## Introduction

Wireless communication is now ubiquitous in our homes, schools, workplaces, and the ambient environment, leading to increased daily exposures to non-ionizing electromagnetic fields (EMF) across all age groups, starting before birth (1–3). All wireless technologies create non-ionizing EMF exposures that are not naturally occurring on Earth (2). Unlike natural EMFs such as the sun and the Earth's geomagnetic fields, with which humans and all living things evolved, anthropogenic wireless signals are complex (4). A radiofrequency (RF) carrier wave is pulsed and modulated to transfer data (video, voice, etc.), creating polarized and highly variable, intermittent signals with lower frequency (ELF/ULF/VLF) intensity variations (3, 5, 6). Environmental levels have increased over the last century, and recent telecommunication upgrades to broadband create a wide swath of simultaneous exposures to multiple modulated frequencies (3). Recent publications emphasize how the complex signaling characteristics (e.g., modulations) of a wireless signal, alongside various environmental and biological parameters, are key to biological impacts (3, 4, 6–8). Most of these factors, however, are overlooked in existing regulations, which focus solely on the carrier wave frequency and power density (8, 9).

For decades, the prevailing assumption underpinning current human exposure guidelines is that because wireless technologies are non-ionizing and lack sufficient energy to break chemical bonds or directly damage DNA, they can only produce harmful effects through heating (thermal) mechanisms (10). This assumption is the basis for the exposure limits of the U.S. Federal Communications Commission (FCC), as well as the Institute of Electrical and Electronics Engineers (IEEE) and the International Commission on Non-Ionizing Radiation Protection (ICNIRP) (10–12). However, this assumption has been roundly challenged by scientific groups such as the International Commission on Biological Effects of Electromagnetic Fields (ICBE-EMF) and others, which argue the ionizing/non-ionizing dichotomy is outdated as adverse biological effects from low-intensity exposures are now well documented (8, 10, 13–19). They conclude that the exposure limits set by the FCC, IEEE and ICNIRP are unable to adequately protect since they are only designed to address the effects of heating from short-term high-intensity exposures, but not for the effects of long-term low-intensity cumulative exposures (8, 10, 13–19).

Numerous studies have reported increased cancer (20–23) and oxidative stress (8, 24, 25) along with genetic (26–28), epigenetic (29–31), reproductive (32–35), endocrine (36, 37), and nervous system (38–42) impacts at exposure levels below the FCC and ICNIRPs heat-based threshold of harm (43–45). Multifrequency (46, 47) and synergistic or combination effects with other environmental exposures, ionizing radiation, chemicals, drugs, and metals have also been reported (48–54). While the FCC, ICNIRP, and IEEE discount EMF non-thermal effects as not established, non-ionizing frequencies are increasingly researched/used in medical applications specifically because of their non-heating effects (55–63).

Furthermore, despite the widespread use of wireless devices by children, FCC limits and test procedures are based on a model of a 220-pound adult male (19, 64), disregarding the unique anatomical and physiological characteristics of children and the developing fetus as well as the increased sensitivity of their rapidly developing brains and bodily systems (19, 65–67). Engineering studies have demonstrated that children's thinner skulls, distinctive physiologies, and more conductive brain tissue result in significantly higher RFR absorption rates, especially in brain regions critical for memory and learning, such as the cerebellum and hippocampus (68–70).

This paper examines the current regulatory framework governing wireless and health, asking:

- What are the standards and guidelines on which FCC regulations are based?
- How did U.S. federal agencies develop the current RFR limit?
- What are the current EMF bioeffect research activities of relevant U.S. federal agencies?
- How have laws and legal decisions shaped governments' response to possible public health impacts?
- Do current practices and agency activities ensure adequate oversight and protection?
- How does the US policy approach compare to other countries?
- Is there evidence of regulatory capture?
- What reforms are needed to address regulatory gaps?

## What are the standards and guidelines on which FCC regulations are based?

FCC human exposure limits for RFR are referred to as thermal limits because they are designed primarily to address the effects of heating. The origin of U.S. thermally based limits dates to Cold War era military research prompted by the development of radar and wireless technologies in WWII (71). In 1953, the Naval Research Laboratory was tasked to investigate radar health effects after the widespread circulation of a Hughes Aircraft Corporation Report by John T. McLaughlin, MD, documenting his observations of cancers, purpura, internal bleeding and other health issues in addition to a “…universal complaint of headaches by personnel working in the vicinity of microwave radiation.” [(72), p. 336]. The report prompted the U.S. Air Research and Development Command and the Navy to convene a series of meetings in which a limit was ultimately adopted based on a calculation by Dr. Herman P. Schwan, a German biophysicist at the University of Pennsylvania who had long worked on the electrical properties of biological materials had come to America under Project Paperclip, a program that brought former German scientists to the U.S. after WWII (71, 73). Schwan recommended 10 mW/cm^2^ (0.01 W/cm^2^) as the critical exposure level based on theoretical calculations of how much heat the body could tolerate and dissipate without significantly increasing body temperature. This threshold was not informed by epidemiological or experimental data regarding health impacts or disease risk. Schwan's limit and the assumptions behind it became the foundation for most subsequent Western standards, including today's FCC limits.

From 1957 to 1960, the Tri-Service Electromagnetic Radiation Bioeffects Program, a joint initiative of the U.S. Air Force, Navy, and Army, conducted an extensive 4-year research effort aimed at investigating microwave health effects (71, 74). Most experiments were of short duration with high intensities (not long-term, low-intensity), observing impacts above 100 mW/cm^2^, confirming the view that non-thermal effects did not exist (72). A safety factor of 10 was incorporated, notably more lenient than the 100- and 1,000-factor safety margins proposed by General Electric and Bell Labs, respectively (75, 76), resulting in a match to Schwan's original recommendation. Although Colonel George M. Knauf, scientific coordinator at the Air Force Rome Air Development Center, stated that the limit had “been arbitrarily established,” [(77), p. 36], he resisted suggestions to use a more stringent limit and concluded that the subsequent Tri-Service research did not “…shake our faith in the validity of this arbitrary level” [(71), p. 1233]. In 1962, Schwan became chair of the United States of America Standards Institute, precursor to the American National Standards Institute (ANSI), C95.1 committee, and proposed that they issue his recommended 10 mW/cm^2^ limit, which they ultimately did in 1966 (71, 78).

Although the 1966 limit was not a formal federal standard, the limit and its updates were used de facto by the military and others for decades despite numerous government reports documenting accumulating scientific research on both heating and non-heating impacts. Dr. Zorach R. Glaser compiled over 2,000 studies reporting behavioral, cardiovascular, neurological, ocular, and endocrine impacts in a 1971 Naval Medical Research Institute (NMRI) Report (79), later updated to over 4,600 studies in a 1977 report which Glaser issued under the National Institute of Occupational Safety and Health (NIOSH) (79–82).

Beginning in 1976 until at least 1987, the Office of Naval Research, along with the National Telecommunications and Information Administration, produced quarterly *Biological Effects of Non-ionizing Radiation* reports, which included the ongoing research conducted in the Soviet Union (83). These publications compiled full research abstracts, including numerous reports of biological effects at non-thermal exposure levels that concluded more research was needed. Soviet literature in these reports described significant neuroendocrine and nervous system impacts as well as cardiovascular alterations, immune system changes, reproductive effects, and behavioral disturbances, many occurring at levels below Schwan's limits.

## How did U.S. federal agencies develop current RFR limits?

The Environmental Protection Agency, with authority regarding ionizing and non-ionizing radiation exposures, has historically played a lead role in this subject. Although the agency was repeatedly tasked with developing federal safety standards for public exposure to RFR over several decades (84, 85), its attempts were unsuccessful, and proposed limits were never finalized (86). This section outlines how U.S. limits were not derived through a public health agency-led risk assessment process but rather by the FCC's adoption of standards developed by military and industry-affiliated groups.

In 1978, the U.S. Comptroller Report recommended that the EPA establish RFR standards highlighting several studies that found biological impacts at low non-thermal intensities, below the 10 mW/cm^2^ ANSI limit, and described how the EPA already had 5 professionals using a mobile van equipped with a measurement system to monitor environmental levels (85).

While the EPA ramped up its research, ANSI and IEEE further developed their standards as detailed in Table 1. Their 1982 limit introduced the whole-body Specific Absorption Rate (SAR), a measure of the rate of absorption into tissue, as the relevant metric (87). The SAR limit of adverse effect was based on the original Tri Service era assumption that heating was the primary and only established effect. As described by the ICBE-EMF in their paper detailing the inaccuracies of the assumptions underlying FCC and ICNIRP limits, “these limits were based on results from behavioral studies conducted in the 1980s involving 40–60-min exposures in five monkeys and eight rats, and then applying arbitrary safety factors…” (10). The behavioral studies measured the internal temperature at which exposed animals stopped food-seeking behavior. Thus, the SAR limit was not based on risk modeling from long-term carcinogenicity or epidemiological health studies.

**Table 1:** Timeline of key RFR exposure standards and reports leading to U.S. guidelines.

**Date**	**Standard**	**Limit**
1966	ANSI C95.1-1966	The first U.S. consensus standard adopted Schwan's recommendation of 10 mW/cm^2^ to address heating impacts.
1971	ANSI C95.1-1971	ANSI retained 10 mW/cm^2^ as an occupation guideline, averaged over 6 minutes. OSHA adopted the ANSI limit, ruled to be advisory only.
1978	U.S. Comptroller Report	The report *More Protection from Microwave Radiation Hazards Needed* recommended that the EPA establish an environmental exposure standard to protect the general public and that the Occupational Safety and Health Administration (OSHA) establish an occupational limit to protect workers based on a current evaluation of scientific data (85).
1982	ANSI/IEEE C95.1 1982	Introduced frequency-dependent limits for power density. The SAR threshold of adverse effects was 4 watts per kilogram (4 W/kg) SAR over the whole body based on animal behavioral disruption studies. A reduction of ten was incorporated to arrive at the limit 0.4 W/kg for whole body SAR. This limit was adopted by the FCC in 1985.
1986	NCRP Report No. 86	National Council on Radiation Protection (NCRP) report, based on literature though 1982, iterated the 4 W/kg thermal threshold but recommended public limits be one-fifth of the 0.4 W/kg worker limit resulting in a.08 W/kg whole body SAR limit. The averaging time was extended from 6 minutes (workers) to 30 minutes for the public. NCRP introduced localized SAR limits for exposure from devices close to the body of 1.6 W/kg for the public and 8 W/kg for workers.
1986	EPA Proposes 4 Options for RFR Limits	The EPA proposed 4 options for RFR limits along with associated financial costs and concluded that adverse human health effects were associated with whole-body average SARs of 1 to 4 W/kg or greater (93, 94). Options 1- 3 were 0.04, 0.08 and.4 W/kg respectively. Option 4 was to develop technical information, not a limit, to control exposure.
1991/1992	ANSI/IEEE C95.1 1992	IEEE introduced controlled (worker) vs uncontrolled (public) limits, still based on the 4 W/kg heating effect threshold. They maintained the.4 W/kg (10x reduction) whole body SAR for workers and set 0.08 W/kg (5x reduction) for public exposure, extending the averaging time from 6 minutes (workers) to 30 minutes for the public. ANSI ratified the limit in 1992 (95).
1993	FCC 93-142 Proposal to Adopt RFR Limits	FCC initiates a Notice of Proposed Rulemaking (NPRM) which recommends using the IEEE C95.1 1992 standard but requests comments on possible alternatives such as NCRPs lower limits for 1.5 to 300 GHz frequencies.
1995	EPA launches renewed research effort toward recommending human exposure guidelines.	The EPA presented a timeline to the FCC on its renewed efforts toward developing a safety limit that addressed both heating effects and modulation, convened a revived interagency workgroup (RFIAWG), and commissioned the NCRP to study the potential role of modulated RFR bioeffects (84, 86). Ultimately, the NCRP study and EPA's efforts were not completed.
1996	FCC Adopts RFR Limits	FCC adopts RFR limits based on both NCRP Report No. 86 and IEEE/ANSI C95.1 1992

Importantly, the ANSI/IEEE 1982 standard noted non-heating related effects at SAR levels below 4W/kg but discounted their relevance. For example, page 13 states, “…modulation-specific effects, such as efflux of calcium ions from brain materials were not considered adverse because of the inability of the subcommittee members to relate them to human health” (87). ANSI/IEEE also noted (page 14), “other characteristics of an incident field such as modulation frequency and peak intensity may pose a risk” and (page 15) that “peak SARs in a biological body can range more than an order of magnitude above the average SAR over a limited mass of the exposed tissue”.

Later that same year, the EPA announced its intention to establish federal RFR limits in the *Federal Register* stating, “The continuing and rapidly increasing number of radiofrequency radiation sources in communications, transportation, defense, industry, consumer products, security, and medical applications has produced a RFR environment to which the entire population is continuously exposed, has increased the magnitude of environmental levels to which members of the public are exposed, and has increased the number of persons exposed to higher environmental levels” [(88), p. 57339].

In 1984, the U.S. Science Advisory Board (SAB), an independent advisory committee to the EPA (89), reviewed the EPA's draft report on biological impacts of EMFs and stated that the report represents an adequate statement of the current scientific literature and can serve as a scientifically defensible basis for the EPA's development of radiation protection guidance (90). The SAB recommendation includes quotes from numerous agencies going back years, requesting the EPA develop human exposure limits. Internal documents indicate EPA's proposed safety limit for exposure was ten times stricter than the ANSI/IEEE 1982 guidelines, but the proposal was never issued due to conflicts within the agency (91).

In 1985, the EPA's Report *The Biological Effects of Radio Frequency Radiation* was released, which concluded a critical level of adverse effects at a SAR threshold of 1 W/kg (92). This threshold is notably lower by a factor of 4 than the 4 W/kg threshold of ANSI/IEEE C95.1-1982, which the FCC adopted as its guideline for new wireless facilities that same year, stating that RFR was a proper environmental concern of the agency (96). In 1986, when the EPA finally proposed possible federal limits, they stated that adverse effects were associated with SARs starting at 1 W/kg, and their research summary highlighted non-thermal effects such as calcium efflux, the potential for cancer promotion, and frequency-specific effects such as changes in brain energy metabolism (93, 94). Ultimately, the agency never issued a decision in the proceeding.

The EPA also never completed another review of the science on the biological effects after its 1984 report. While fully reviewing the intertwined policy history of powerline frequency EMF and magnetic fields is beyond the scope of this paper, it is important to note that, like RF, federal agencies never developed a safety standard for these lower frequency non-ionizing exposures. Reports on non-ionizing EMFs were drafted and redrafted but never finalized (97, 98). In 1990, the EPA released a draft report that recommended designating powerline-frequency EMFs as “probable human carcinogens” and RFR as a “possible human carcinogen” [(97), p. 1]. This conclusion was deleted from the subsequently released draft, which stated it was inappropriate to compare EMFs to chemical carcinogens and EMFs were a possible, but not proven, cause of cancer in humans (99). The EPA Science Advisory Board (SAB) and federal agencies repeatedly reviewed the draft, but it was ultimately shelved (100).

All the while, the U.S. stayed up to date on the ongoing research, including that of the Soviet Union and Eastern Europe. The 1988 Air Force Report on the biological effects of RFR includes an overview of research that found cardiovascular, immunological, and hematological effects, stating, “…exposure to RF/MW radiation is known to have a biological effect on animals and humans. Damage to major organs, disruption of important biological processes, and the potential risk of cancer represent the dangers of RF/MW radiation to living organisms. Pulsed radiation appears to have the greatest impact on biological materials” [(101), p. 2]. Yet in 1991, when IEEE revised the ANSI limits and introduced limits for the general public (see Table 1), they argued against a larger limit reduction for the general public because “no reliable scientific data exists” showing certain population subgroups are more at risk or that duration of exposure can impact risk (95). Again, they stated that non-thermal and modulation-specific effects were not meaningfully related to human health.

**Figure 1:**
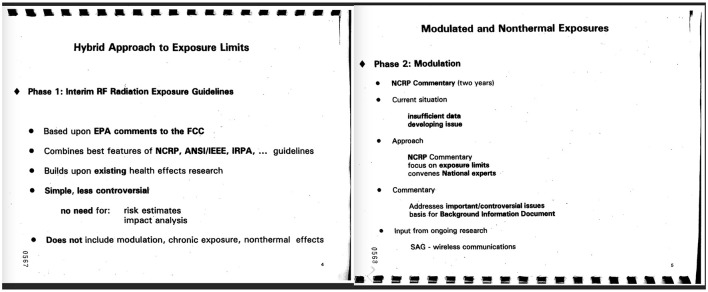
1995 briefing for the FCC by the EPA on the development of RFR exposure guidelines (102). Figure from U.S. government document in the public domain (102).

Importantly, the EPA stated in 1995 that it was poised to issue RFR limits (102). As shown in the EPAs presentation (Figure 1), Phase 1 would address thermal effects (understood as not including modulation or chronic exposure), and Phase 2 would address biological impacts of modulated and non-thermal exposures. However, again, EPA's work on developing safety limits was halted. That year (1995) the Senate Committee on Appropriations cut $350,000 from the EPA EMF budget, because “the committee believes EPA should not engage in EMF activities” [(103), p. 5]. The U.S. House of Representatives also cut EMF programs. In addition to the budget cuts, several staffers reportedly blamed the dismantling of the research program on senior officials for not wanting to revisit such a controversial subject (98), some of whom then went on to work for the telecommunications industry (104).

### FCC promulgates human exposure limits in 1996

In 1996, with the EPA's standards development shuttered, the FCC adopted limits (Table 2) (9, 105) based on recommendations from the ANSI/IEEE C95.1-1992 standard (95) and the NCRP 1986 Report (106), which are both based on the 4W/kg SAR heating threshold of harm identified in acute animal studies conducted in the 1980s and earlier (10). Thus, FCC limits are not federally established, health-protective safety standards for long-term exposure, as they were not developed through a formal hazard/risk assessment that evaluated toxicity and dose response by health agencies (107).

**Table 2:** FCC general public (A) and occupational (B) limits for RFR: SAR limits and maximum permissible exposure (MPE) limits.

**A. FCC limits for general public/uncontrolled exposure to RFR**		
**Specific absorption rate (SAR) limit**		
**Whole body SAR**	**Local SAR head and trunk**	**Extremity local SAR** **hand, wrist, feet, ankle, pinna (outer ear)**
0.08 W/kg Averaged over whole body	1.6 W/kg averaged over 1 gram cube of tissue	4.0 W/kg averaged over a 10-gram cube of tissue
**Maximum public exposure (MPE) limit**				
**Frequency range (MHz)**	**Electric field strength (V/m)**	**Magnetic field strength (A/m)**	**Power density (mW/cm** ^2^ **)**	**Averaging time (minutes)**
0.3–3.0	614	1.63	(100)	30
3.0–30	842/f	2.19/f	(180f^2^)	30
30–300	27.5	0.073	0.2	30
300–1,500			f/1,500	30
1,500–100,000			1.0	30
General public limits apply in situations in which the general public may be exposed, or in which persons that are exposed as a consequence of their employment may not be fully aware of the potential for exposure or cannot exercise control over their exposure. Public limits are used for consumer devices such as cell phones (9, 105). f, frequency in MHz.				
**B. FCC limits for occupational/controlled exposure to RFR**		
**Specific absorption rate (SAR) limit**		
**Whole body SAR**	**Local SAR; head and trunk**	**Extremity Local SAR; hand, wrist, feet, ankle, pinna (outer ear)**
0.4 W/kg; averaged over whole body	8.0 W/kg; averaged over 1 gram cube of tissue	20.0 W/kg; averaged over 10 grams of tissue
**Maximum public exposure (MPE) limit**				
**Frequency Range (MHz)**	**Electric Field Strength (V/m)**	**Magnetic Field Strength (A/m)**	**Power Density (mW/cm** ^2^ **)**	**Averaging Time (minutes)**
0.3–3.0	614	1.63	(100)	6
3.0–30	1,824/f	4.89/f	(900/f^2^)	6
30–300	61.4	0.163	1.0	6
300–1,500			f/300	6
1,500–100,000			5	6
Occupational limits apply when exposure occurs as a consequence of employment provided the individual is “fully aware” of the potential for exposure and can exercise control over the exposure. “Fully aware” means the person has received written and/or verbal information explaining the potential for RFR exposure. With the exception of transient persons, “fully aware” also means the person has received appropriate training regarding work practices relating to controlling and mitigating exposure. Occupational limits also apply where exposure is of a transient nature as a result of incidental passage through a location where levels may be above general population limits, as long as the exposed person has been made fully aware of the potential for exposure and can exercise control over his or her exposure by leaving the area or by some other means (9, 105). f, frequency in MHz.				

MPE limits are frequency-dependent and are generally used for compliance related to the ambient environmental levels created by cell towers and wireless network infrastructure. “Portable devices” are defined as devices (such as cell phones) used within 20 cm of the user's body, and they require compliance with local SAR limits for 100 kHz to 6 GHz and power density limits for frequencies above 6 GHz.

### The National Toxicology Program animal studies

The U.S. Food and Drug Administration (FDA), aware of the data gaps regarding long-term, chronic exposures, nominated the National Toxicology Program (NTP) under the National Institute of Environmental Health Sciences (NIEHS) to conduct large-scale animal research on carcinogenicity in 1999 because “…the existing exposure guidelines are based entirely on protection from acute injury from thermal effects of RFR exposure and may not be protective against any non-thermal effects of chronic exposures” [(108), p. 2].

The ensuing NTP research effort spanned decades. As presented in the 2008 U.S. Congressional (109) and 2009 U.S. Senate (110) subcommittee hearings on cell phone radiation health effects, the NTP built special chambers and initiated large-scale animal studies exposing rats and mice to cell phone radiation for up to 2 years. In 2018, the NTP final report concluded significantly increased gliomas of the brain and schwannomas of the heart in male rats (111). An expert peer review panel concluded the schwannoma data indicated “clear evidence” of an association between RFR and cancer. Additionally, DNA damage, reduced pup birth weights, and the induction of cardiomyopathy of the right ventricle of the heart were observed (111, 112). Notably, when New Hampshire 5G Commissioner Denise Ricciardi asked, “You found damage, so doesn't that mean that FCC's assumption that only heating can cause damage is incorrect and no longer accurate?” NTP's scientist Michael Wyde replied, “A lot of people believe unless you heat tissues, you won't see health effects with RF. This study disproves that, as we did not have overheating, but we did see damage” [(113), p. 139].

However, the FDA disagreed with the conclusions and repeatedly issued statements discounting the animal study's relevance to humans (114, 115) despite NTP's own experts maintaining that the findings were relevant to human health assessment (19, 112, 116). Further, the FDA did not release any reports providing a quantitative risk assessment on the NTP study data to inform an understanding of the implications for human health. Published analysis of the NTP data by non-government U.S. scientists concluded that FCC human exposure limits should be strengthened by at least 200-to 400 times to protect children (45).

Nonetheless, in 2019, the FCC terminated its 7-year inquiry, which had asked if the 1996 limits needed to be revised (117). Referencing the FDA's dismissal of the NTP findings, the agency announced the 1996 limits were adequately protective, stating, “We take to heart the findings of the Food & Drug Administration (FDA)... that the weight of scientific evidence has not linked cell phones with any health problems” [(172), p. 2]. The IEEE and ICNIRP subsequently issued revised RF-EMF exposure limit recommendations, discounting the NTP study findings and reiterating that acute heating effects were the only established harm (11, 12).

Although the NTP later initiated new studies to investigate the DNA damage they had observed (118), in 2024, they stated that these follow-up studies had ceased because it was “technically challenging and more resource-intensive than expected” (119). They released a 2025 report documenting a lack of effects in one completed 5-day pilot study but state that, “high-quality studies to understand the effects of RFR exposure on biological responses are needed given the widespread human exposure to RFR associated with cell phone use” (120). Despite the 5.3-million-dollar price tag, the exposure system for the follow-up research was “disassembled following completion of the studies, and the chambers are not available for other scientists” (121).

## What are the current EMF bioeffect research activities of relevant U.S. federal agencies?

While the push to fast-track wireless development is framed as a geopolitical race with China and Russia (122), these nations, along with the European Union, India, and numerous other governments, at least fund research programs studying health and wildlife impacts of wireless technology (123–126). Absent from the U.S. is a parallel effort to ensure rigorous science-based evaluation (127). As detailed in Supplementary material 1, U.S. agencies, defunded years ago, lack current research activities and administer little oversight, allowing industry to successfully use the race narrative to support deregulation efforts at the federal, state, and local levels (128).

Supplementary material 1 lists details the near absence of current EMF bioeffect research activities by key U.S. agencies and entities including the Environmental Protection Agency (EPA), National Institute for Occupational Safety and Health (NIOSH), Department of Labor Occupational Safety and Health Administration (OSHA), National Cancer Institute (NCI), Centers for Disease Control and Prevention (CDC), Food and Drug Administration (FDA) and the U.S. Fish and Wildlife Service (USFWS), the U.S. Radiofrequency Interagency Work Group (RFIAWG), FDA's Technical Electronic Product Radiation Safety Standards Committee (TEPRSSC) as well as the recent directive that Health and Human Services (HHS) report on knowledge gaps.

Notably, in 1999 and 2003, federal experts of RFIAWG issued a critique of federal limits as “outdated and insufficiently protective,” highlighting 17 critical issues including the lack of biological basis, inadequate dosimetric modeling, failure to address modulated exposures, flaws in the two-tier occupational/public system, insufficient attention to long-term health effects and problems with how averaging over time and tissue volume can mask peak exposures. They called for a comprehensive scientific review with evidence-based standards to better protect public and worker health (129, 130).

While most U.S. federal agencies maintain web content with broad, often dismissive statements about health risks, none have issued formal health risk evaluations evaluating the totality of current scientific evidence. Although the FDA did release a literature review (131) dismissing the NTP findings, its conclusions of safety were roundly criticized on several counts, including a lack of adherence to quantitative risk assessment methodology (132). Further, the report was narrowly focused and confined to cell phones and cancer, omitting all consideration of non-cancer health endpoints and of effects from environmental exposures. No health agency other than the FDA has issued any type of report attempting to review the issue since the 1986 EPA Report. Mouzaffar states that “the FCC and FDA have failed in their obligation to prescribe safe RFR guidelines produced from wireless communication devices to protect the public health and safety” [(133), p. 227].

With NTP research halted, the only ongoing studies appear to be conducted within the Department of Defense. James Lin, former ICNIRP Commissioner, states that the recent research at the Air Force RF Bioeffects Branch and the Defense Advanced Research Projects Agency (DARPA) represents a “paradigm shift” into exploration of non-thermal impacts (134). Their studies utilizing FCC-compliant exposures have reported epigenetic impacts and brain pressure levels from pulsed microwaves comparable to football injuries (31, 39). In 2019, the Air Force Radio Frequency (AFRF) Bioeffects Branch awarded a $30 million contract to General Dynamics Information Corporation (135) with the initial solicitation stating that research results would be utilized for national and international health and safety standards and used for Air Force occupational safety, as well as to support the development of directed energy technologies (136, 137). In 2025, another solicitation was posted at $39.7 million (138). However, there is no evident coordination with non-civilian agencies, as the Air Force did not submit comments to the FCC in its RF-related proceedings (117).

### The World Health Organization (WHO)

With federal reports lacking, the WHO is often referenced as an authority. However, the existence of two separate entities housed under the WHO umbrella, the EMF Project and the World Health Organization's International Agency for Research on Cancer (WHO/IARC) has complicated the public and policymakers' understanding of the WHO's conclusions on RFR safety.

In 2011, the WHO/IARC classified wireless RFR as “possibly carcinogenic” (Group 2B) (139). Their advisory group has, in 2019 and again in 2024, recommended wireless RFR be re-evaluated as a high priority (140, 141) due to the epidemiological and experimental animal studies published since 2011. Several analyses, disagreeing with the ICNIRP, IEEE and FDA's dismissal of the NTP, conclude that the animal experiments of the NTP, along with those of the Ramazzini Institute in Italy [which used much lower wireless exposures than the NTP to mimic cell tower environmental levels (45)] have strengthened the evidence base and could justify the WHO/IARC upgrading the classification of RFR to a “probable” or even to “known” human carcinogen (16, 20, 23, 112, 142, 143). However, as of this writing, the WHO/IARC has yet to announce a date to convene expert working groups on RFR.

In contrast to the WHO/IARC, which removed scientists from its working group in 2011 due to conflicts of interest (144), the EMF Project has been criticized for its longstanding ties with industry and ICNIRP (76, 145–147). Michael Repacholi, the first chairman of ICNIRP, suggested that the WHO start the EMF Project in 1995, and he arranged for a large part of its financing to be from the telecommunications industry (76, 145).

The FCC referenced the WHO EMF Project webpages in support of its 2019 decision that its RFR limits were adequate (148). However, at that time, the EMF Project's last completed review on RFR health risks dated to 1993 (149). More recently, the WHO EMF Project has commissioned a series of systematic reviews on several health endpoints (150). While most of the reviews concluded the evidence did not show adverse effects and that more research was needed, these reviews have been sharply criticized by independent scientists (134, 151–156) for “numerous flaws, significant methodological concerns, as well as likely biases” which “undermine the validity of the authors' conclusions and raises serious doubts about the suitability of most of these reviews for informing policy or risk management decisions (156). While one or more members of ICNIRP were involved in each systemic review (156), none of the WHO-commissioned review scientific teams included any of the over 250 scientists who signed the EMF Appeal, a petition to the United Nations by international scientists calling for stronger protections from non-ionizing EMFs (15, 157). However, safety cannot be assured from these reviews as the WHO-commissioned review on animal experimental evidence and cancer did conclude that there was “high certainty” evidence associating RFR with two tumors, gliomas and schwannomas, also reported in human studies of long-term cell phone use (158).

Further, while the WHO commissioned reviews are referenced as “one of the most comprehensive evaluations of environmental health evidence to date” [(150), abstract], they did not include reviews focused on endpoints such as genotoxicity, synergistic effects, and impacts to the immune and endocrine systems.

## How have laws and legal decisions shaped governments' response to possible public health impacts?

Section 704 of the Telecommunications Act (TCA) of 1996 prohibits state and local governments from regulating cell tower placement based on “environmental effects” of RFR emissions, as long as emissions comply with FCC limits (159). This provision has been interpreted by some courts as a federal preemption, allowing it to serve as both a regulatory safe harbor, shielding the industry from liability for health-related damages, and a de facto gag rule, silencing public discussion and allowing industry to threaten lawsuits when health is raised during public hearings/siting decisions (159–161). Before the passage of the TCA, many state and local authorities had adopted ordinances that restricted the number and location of wireless towers and base station antennas due to health concerns (160). But now these governments, which have a statutory responsibility to protect the health, safety, and welfare of citizens, have been largely stripped of their ability to protect schools, homes, and wildlife areas.

The situation is a legal quagmire as federal agencies have failed to ensure up-to-date, rigorous scientific reviews, yet policymakers are effectively barred from voicing concerns that risk could exist as federal regulations are outdated. Section 704 not only prevents most decision-makers from expressing health-related justification for distancing cell towers from schools and homes, but it also denies judicial remedies and due process, offering no avenue to mitigate risk or any avenue to address harm (159–161).

Section 704, as applied, transgresses several constitutional issues, many of which have been raised in legal challenges over the years without avail due to one primary early ruling, *Cellular Phone Taskforce v. FCC* (162). In this case, filed just after the FCC promulgated its 1996 limits, numerous groups and petitioners argued that the FCC's RFR limits failed to account for evidence of non-thermal health effects. In 2000, the Court sided with the FCC, enshrining deference to its federal authority, based on the assumption that the FCC “could reasonably” expect agencies such as the EPA “to keep abreast of scientific developments in carrying out their missions” (162). The *Cellular Phone Taskforce* case became the cornerstone for all subsequent telecom cases when courts routinely deferred to the FCC authority regarding RFR emissions.

In the subsequently filed Petition for Writ of Certiorari, the U.S. Supreme Court was asked to review the decision by the petitioners' attorneys, including Whitney North Seymour, Jr. a former New York State Senator, former U.S. Attorney for the Southern District of New York and co-founder of the Natural Resources Defense Council (163). The petition highlighted the lack of federal agency activities as the central material error in the Second Circuit court's ruling that begs for re-argument today asking:

“Where Congress has failed to fund continuing Federal research into potential adverse health effects of radiation emission from cellular phone towers, does Congress have the power to prohibit State and local governments from protecting the health of their citizens by taking into account available research and official standards from other countries in regulating the placement of cellular phone towers?” [(163), p. 1]

The Petition argued that:

“…dated, inappropriate research, reviewed by authorities from inappropriate professions, is being used to reach conclusions about public safety concerning radio frequency radiation. Why has there been no better updating of the FCC standards? The answer is obvious: because Congress has not appropriated adequate funds to conduct non-thermal research by the key federal agency—EPA.”

“The Court's reliance on the EPA was technically correct but substantively naive. What the Court did not realize was that Congress terminated funding for radiation research by EPA in 1996, and no staff has been available at EPA to conduct such research for the past five years” [(163), p. 14].

In other words, the FCC did not have adequate expertise from the appropriate government agency to rely on regarding health effects from ambient RFR exposures. The Supreme Court did not take up the case in 2000. But now, over two decades later, the petition's arguments are even more relevant: no independent federal agency with environmental or health expertise is actively monitoring RFR risks from cell towers, and local governments remain hamstrung, fearing industry lawsuits should they speak on the issue of health effects in infrastructure siting decisions. While the FCC stated in its 2013 Inquiry, “…since the Commission is not a health and safety agency, we defer to other organizations and agencies with respect to interpreting the biological research necessary to determine what levels are safe” [(164), p. 3], there is no federal agency to defer to. This situation is more germane than ever for another legal challenge.

### Federal court mandates FCC provide rationale for maintaining 1996 RFR limits

The second major challenge to FCC limits came after the agency decided to maintain its 1996 limits in 2019. In 2021, the U.S. Court of Appeals for the D.C. Circuit ruled in *Environmental Health Trust et al. v the FCC* (see Table 3) that the FCC's decision was “arbitrary and capricious,” citing the agency's failure to address evidence submitted to the agency reporting a myriad of harmful effects at levels permitted under current regulations [(165), p. 9]. The FCC was ordered to explain how its limits were adequately protective on six issues: non-cancer health impacts, long-term effects, children's vulnerability, environmental impacts, compliance test procedures, and the significant technological changes/advances since 1996. As detailed in Table 3, as of this writing, the FCC has not responded to the court's mandate.

**Table 3:** Timeline: Environmental Health Trust et al. v the FCC lawsuit on U.S. human exposure guideline inquiry.

**Year**	**Event/action**	**Description**
2012	GAO Report: “Exposure and Testing Requirements for Mobile Phones Should Be Reassessed” (171)	The Government Accountability Office (GAO) report recommended that the FCC formally reassess and, if appropriate, update its exposure limits and cell phone SAR test requirements stating: “The FCC RF energy exposure limit may not reflect the latest research, and testing requirements may not identify maximum exposure in all possible usage conditions” [(171), Highlights p. 1].
2013	FCC Opens Inquiry on RFR Exposure Limits (ET Docket No. 13–84) (117)	The FCC Inquiry requests comment on whether its 1996 RFR human exposure limits and policies should be reassessed. In summary: •Are the 1996 exposure limits appropriate to protect public health considering recent research? •Are additional exposure reduction and precautionary policies needed, especially regarding children? If so, would they be useful or counterproductive. •Should premarket SAR evaluation procedures require direct body contact positions? •Are SAR limits protective regarding metal implants, eyeglasses, jewelry, or metallic accessories. •Should the FCC require more disclosure around RFR exposure levels “perhaps in manuals, at point-of-sale, or on a website.” •Should more consumer information on RFR exposure (on health, reducing exposure, and base station exposure) be provided to the public?
2013 to 2019	Hundreds of comments submitted to the FCC record in its inquiry (ET Docket No. 13–84)	Comments on the FCCs RFR Inquiry were submitted from scientific, medical and public health organizations, local governments, school boards, researchers, medical practitioners and the public.
2019	FCC decides to maintain 1996 limits and proposes new limits for ranges not previously regulated (172)	The FCC decides to maintain its 1996 limits without further review stating there is “no appropriate basis” to revise its RFR exposure limits, additional precautionary policies are not indicated and that revising SAR tests to require body contact positions is not necessary. The FCC also proposed expanding the range of human exposure limits to include new frequencies: •300 kilohertz (kHz) down to 3 kHz, with sources such as wireless power transfer in electric vehicles. •100 GHz up to 3 THz for future technologies. Comments on the newly proposed limits remain open under Docket 19-226.
2020	14 petition for judicial review (173)	Petitioners argue that the FCC decided its limits did not need to be revised without meaningfully addressing record evidence of cancer, neurological impacts, reproductive harm, electromagnetic hypersensitivity (EHS), children's vulnerability, wildlife impacts and how the premarket SAR tests are outmoded. Petitioners Environmental Health Trust (EHT), Consumers for Safe Cell Phones, Elizabeth Barris, and Theodora Scarato filed in the DC Circuit. Petitioners Children's Health Defense (CHD), Michelle Hertz, Petra Brokken, Dr. David Carpenter, Dr. Toril Jelter, Dr. Paul Dart, Dr. Ann Lee, Virginia Farver, Jennifer Baran, and Paul Stanley filed in the Ninth Circuit. CHD's case was consolidated with EHT's in the DC Circuit.
2020	Amicus briefs filed (174–177)•Natural Resources Defense Council •Joseph Sandri with Declaration of Dr. Linda Birnbaum •Dan and Catherine Kleiber •Building Biology Institute
2021	U.S. Court of Appeals for the D.C. Circuit ruled in EHT et al. v FCC (165)	On August 13, 2021, the DC Circuit ruled the FCCs decision to maintain its 1996 limits as “arbitrary and capricious in its failure to respond to record evidence that exposure to RF radiation at levels below the Commission's current limits may cause negative health effects unrelated to cancer” [(165), p. 9]. and mandated that the FCC provide a reasoned explanation for how its limits were adequately protective regarding: •Children's health •Health implications of long-term exposure •Impacts to the environment •Cell phone and wireless device compliance test procedures •The ubiquity of wireless devices, and other technological developments since 1996 The Court noted the lack of meaningful comments from other federal agencies stating on page 15 and 16: “The silence of other expert agencies, however, does not constitute a reasoned explanation for the Commission's decision to terminate its notice of inquiry for the same reason that the FDA's conclusory statements do not constitute a reasoned explanation: silence does not indicate why the expert agencies determined, in light of evidence suggesting to the contrary, that exposure to RF radiation at levels below the Commission's current limits does not cause negative health effects unrelated to cancer. Silence does not even indicate whether the expert agencies made any such determination, or whether they considered any of the evidence in the record.” As the FCC did not appeal to the U.S. Supreme Court, the decision stands.
2025	No response from the FCC to the Court mandate as the date of this publication.	

In their decision, judges highlighted how the FCC erroneously presented the “silence” of federal agencies as supporting the FCC's limits despite no agency having submitted a substantive, up-to-date science-based assessment (165). Although the FCC had requested input, agencies such as the EPA, National Cancer Institute, and National Toxicology Program (NTP) offered only cursory comments, and the Department of Labor acknowledged its lack of formal risk assessment (166–170).

In addition, the court noted how the FCC had over-relied on the FDA's one-paragraph conclusion (165, 169). The limited scope of the FDA's literature review (focused only on pre-5 G cell phones and cancer) renders it inapplicable to the court's concerns over long-term health effects from the ambient environmental exposures from cell tower networks, 5G's higher frequencies, wildlife impacts, and any studies of reproductive, neurological, and endocrine effects. While many of these issues would fall under the EPA's purview, the agency, defunded for ambient RFR exposure oversight, only submitted a two-sentence comment to the FCC (170) and did not respond to the FCC's request for a more substantive comment.

## How does the U.S. policy approach compare to other countries?

**Figure 2:**
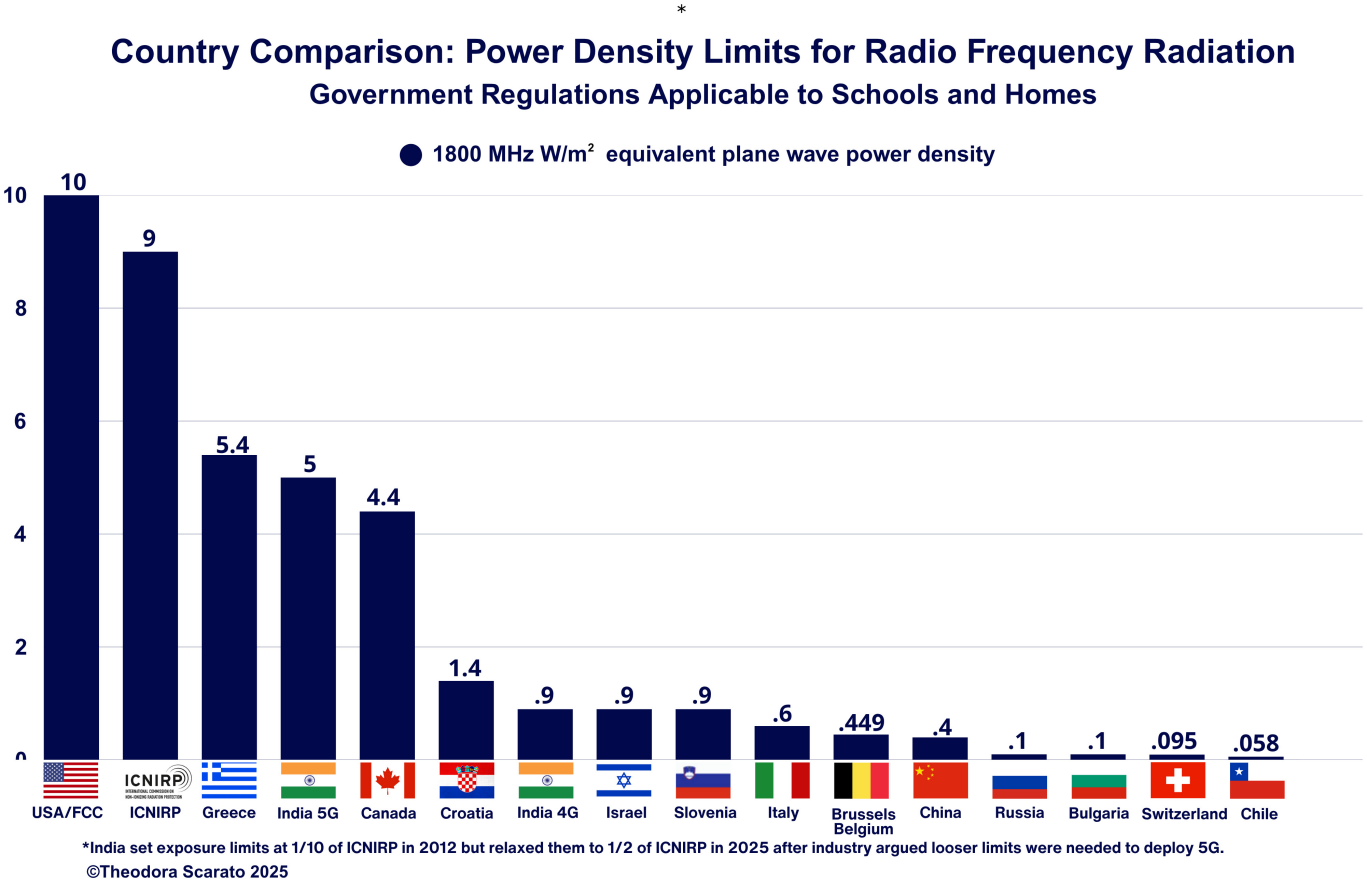
Comparing the U.S. to countries with more stringent RFR limits.

The lack of regulatory updates has resulted in the US maintaining among the most lenient limits worldwide for environmental exposures, as detailed in Figure 2. The U.S. maintains a one-size-fits-all approach without additional protections for sensitive areas, such as schools, daycares, and homes. While many countries have adopted the thermally based limits of ICNIRP, several nations have implemented more stringent thresholds based on their scientific research or precautionary frameworks that attempt to mitigate risk. Graphic 1 shows the selected country's general public limit for 1,800 MHz W/m^2^ equivalent plane wave density specifically applicable to schools and/or homes (178–184). Switzerland and Italy's limits in Figure 2 apply to places of “sensitive use” such as apartment buildings, schools, hospitals, permanent workplaces, children's playgrounds, and where people stay for hours. Biologically relevant changes have been documented at very low intensities, even lower than countries' more stringent limits (10, 16–18, 22, 26–28, 31, 44, 51, 53, 185–187).

Russian research on electromagnetism, dating to the 1840s, has long recognized non-thermal impacts, and its scientists have consistently recommended limits to address their observed biological impacts to the central nervous system (182). China also has active ongoing bioeffect research, and its scientists cited studies showing immune, behavioral, neurological, and reproductive impacts at levels below the 4 W/kg SAR heat threshold as the rationale for its tighter limits (46, 47, 188). The regulatory landscape is always shifting, and several nations, such as Poland, Belgium, India, and Italy, relaxed their rules under pressure from industry, which claimed strict limits would hinder 5G deployment.

In addition, in contrast to the U.S., countries have enacted various policy measures to provide oversight, transparency, and mitigate risk as presented in Table 4 (19, 179, 181, 184, 189–193).

**Table 4:** Examples of international policies on wireless radiation to mitigate risk and ensure compliance.^*^

**Action, law, policy or directive**	**Country examples**
**Public health recommendations**	
Health agency public health advice that people should reduce exposure to cell phone RF radiation, especially for children.	United Kingdom, Russia, Switzerland, Finland, Ireland, Germany, Belgium, Greece, Israel, Turkey, Singapore, France, Denmark, India, Austria, Cyprus, Canada, Italy, French Polynesia, Republic of Korea, Sri Lanka, Croatia, Ivory Coast, European Parliament Resolution 1815
Public education campaign and/or a website that recommends and informs people how to reduce exposure.	France, French Polynesia, Israel, Italy, Cyprus, Tunisia, Switzerland, Belgium, Republic of Korea, Ivory Coast
Public health guidance to prefer wired over Wi-Fi in kindergartens and schools.	France, Israel, French Polynesia, Russia, Cyprus
**Laws or policies to reduce exposures**	
More stringent limits for environmental exposures from cell towers and wireless networks and/or policies to reduce levels below ICNIRP/FCC limits due to either a lower national limit or additional measures in “sensitive areas” (e.g., schools, daycare centers and hospitals).	Italy, India, Israel, Chile, Croatia, Ukraine, Kuwait, Greece, China, Russia, Canada, Switzerland, Belgium, Bosnia Herzegovina, Grand Duchy of Luxembourg, Greece, Belarus, Georgia, Serbia, Slovenia, Montenegro, Croatia, Bulgaria, Turkey Liechtenstein, Tajikistan, Kazakhstan, Uzbekistan, Kyrgyzstan, Moldova, Kuwait, Republic of Moldova, Iraq
Advertising mobile (cell) phones to young children prohibited	France, Belgium, French Polynesia
Sale of phones designed for young children prohibited	Belgium, France, French Polynesia (Minister of Health is authorized to ban)
Wi-Fi prohibited in daycares and kindergarten	France, Israel, French Polynesia (unless for specific activity), Cyprus (Directive)
Wi-Fi off or minimized in elementary classrooms	France, Israel, Cyprus (Directive)
**Monitoring and measuring programs**	
Measurement program to monitor nationwide environmental levels from cellular networks and ensure compliance.	France, Greece, Turkey, Romania, Serbia, India, Israel, French Polynesia, Croatia, Bulgaria, Tunisia, Malta, Brazil, Bahrain, Monaco, Bhutan, Senegal, United Kingdom, Australia, Spain, Austria, India, Israel, Gibraltar, Brussels Belgium, Switzerland, Norway, Lithuania
Post market surveillance program tests cell phones for SAR compliance.	France, Canada
**Transparency measures**	
Required labeling of cell phone radiation SAR levels on product packaging and/or point of sale.	France, Israel, India, Belgium, Russia, Republic of Korea, China
RF-EMF environmental level measurements from cell towers and base stations are publicly shared.	France, Greece, United Kingdom, Australia, Austria, Gibraltar, Belgium, Switzerland, Tunisia, Bahrain, Monaco, Senegal, Spain, Israel, Malta, Brazil

^*^This list is not based on a systematic review, and additional countries may also have relevant policies not included in this table.

## Do current practices and agency activities ensure adequate oversight and protection?

In addition to the near absence of civilian bioeffects research, the U.S. lacks numerous other regulatory elements related to health, safety, oversight, and enforcement.

### No premarket safety testing

Unlike the process for introducing new drugs, the U.S. lacks a process that requires premarket safety testing for health effects before new RF/EMF technologies are introduced. Thus, the FCC approved deployment of millimeter-waves and sub-terahertz frequencies (194, 195) for 5G and future networks and the agency continues to allow new technologies to be deployed despite research indicating heightened rates of absorption into the skin (196, 197) as well as into insects' bodies (198, 199). The FCC's 2019 rules fast-tracked the deployment of small cells, stating that 800,000 new wireless sites in the U.S. were needed for the 5G network, despite the reality that no agency with health or environmental expertise had ongoing research activities to ensure the safety of ambient exposures.

### No post-market medical or health surveillance

There is no post-market medical or health surveillance system. The public, physicians, and environmental experts lack access to a formal reporting mechanism to document symptoms, diagnoses, or observed ecological changes following RFR exposure. No public database exists to track and analyze these incidents. The absence of health and environmental surveillance hinders the identification of patterns, allows early warning signals to be missed, and prevents data-driven policy.

### Outdated premarket RFR compliance tests

New phones and wireless devices can be brought to market so long as they comply with the FCC's 1996 local SAR limits. While the U.S. head/body SAR limit (1.6 W kg) is more stringent than the ICNIRP limit (2.0 W/kg), the use of a heat-based limit as well as the lenient separation distances permitted in compliance test procedures, invalidates safety comparisons. A summary of just a few of the numerous problems identified in premarket SAR tests are presented in Table 5, with more comprehensive critiques in references (10, 18, 19, 129, 130, 200).

**Table 5:** Selected critiques of SAR metric and SAR/SAM test regarding adequacy for health protection.

**Element**	**Practice/procedure**	**Critique**
SAR limit	Limit is based on heating impacts.	Studies used to identify heat threshold for limit are animal studies from early 80s and 70s with small sample sizes and under 50-min exposures (10). Limit is not based on biological impacts from non-heating intensities or chronic exposure.
SAR “safety” margin	FCC claims a large safety factor exists.	The reductions were only applied to the heat-based threshold. Arbitrarily selected uncertainty/safety factors are inadequate for public health protection and are not consistent with the approaches used by public health agencies (10).
SAR measurement of rate	SAR does not measure total dose.	SAR values are a measurement of the rate of energy absorption per a specified mass of tissue. SAR values do not reflect the total absorbed dose or cumulative exposure, unlike the metrics used in ionizing radiation limits (201). Thus, exposure is allowed to continue indefinitely so long as the rate remains within SAR limits, omitting consideration of total dose over time.
SAR averaging	SAR rate is calculated by averaging over tissue.	Averaging measurements over a volume of tissue (1 or 10 grams) obscures peak levels absorbed by specific tissues/organs (129, 130).
SAR test phantom	SAR tests use a 220-pound adult male SAM body phantom.	SAM does not ensure conservative evaluation of all users/bystanders' exposures (19, 69, 202, 203). Engineering studies have also demonstrated that children's thinner skulls, distinctive physiologies, and more conductive brain tissue result in significantly higher RFR absorption rates, especially in brain regions critical for memory and learning, such as the cerebellum and hippocampus (68–70).
SAR test phantom materials	SAM phantoms are a plastic shell filled with homogenous liquid of salt, sugar and surfactants.	Human brain and body tissue is not homogenous. The continued sole use of SAM, when more precise methods are available, ignores the complexity of human tissue as well as the unique anatomical characteristics of children and the developing fetus, disregarding the increased sensitivity of their rapidly developing brains (19, 40, 68–70).
Body SAR test usage positions	Rules allows separation distance between the phone and body.	FCC's authorized test positions do not reflect today's real-world usage scenarios. Although people use phones tucked in tight pockets or pressed against their chest and abdomen, manufacturers have long been allowed to use a separation distance, from 5 to 25 mm for body SAR compliance tests.
SAR test set up	SAR tests only one device at a time.	People are exposed to numerous devices and networks all at once (from cell phones to Wi-Fi routers/printers, wireless wearables, “smart” devices, cell towers, small cells, etc.), which can increase their total exposure (204–206). Premarket tests do not account for total real-world combined overlapping exposure.
SAR test environment	Laboratory tests omit real world parameters.	Various parameters omitted from FCC authorized tests can impact a person's exposure. For example, metal inside (implants), on (jewelry/piercings) or near the body (usage in trains, cars etc.) can significantly increase exposure (207–212).

### The FCC's 2 mm SAR tests

**Figure 3:**
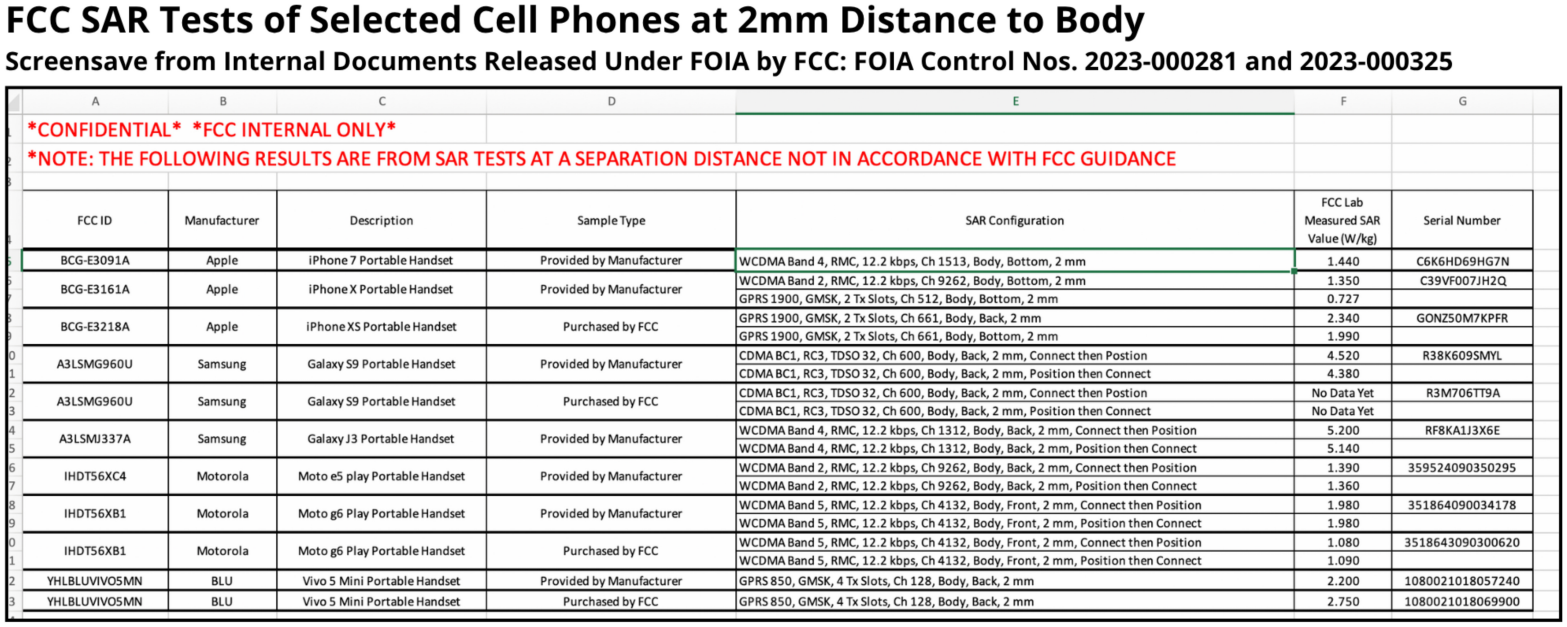
FCC SAR tests at 2 mm distance find SAR values that exceed FCC's limit. Figure from U.S. government document in the public domain (217).

Cell phones are not required by the FCC to be tested in direct body contact positions because the test procedures are based on pre-1996 usage scenarios when phones were worn on belt clips. French government and independent investigations have documented that cell phones can exceed exposure limits when tested in real-world direct body contact scenarios (213, 214). In 2019, the authors' Freedom of Information (FOIA) request found that when the FCC tested cell phones at 2 mm from the body, mimicking a phone in the pocket, they measured SAR levels that exceeded the FCC's limit (215–217). Figure 3 presents the FCC's spreadsheet of its 17 body SAR measurements for 11 cell phones that the agency tested in the summer of 2019. Although the FCC's body SAR limit is 1.6 W/kg, 10 of the 17 FCC laboratory measurements exceeded this limit, one model by up to 325%.

If the FCC had tested the same phone models at 0 mm, mimicking direct body contact positions, the SAR levels likely would exceed FCC limits even more significantly, as conservative estimates indicate a 15% SAR increase for each mm of distance (213). The FCC FOIA data also highlights the variability in SAR measurements and +/−30% margin of error (or uncertainty factor) allowed by the FCC (218), as the same models had different SAR values depending on whether the phone was provided by the manufacturer or purchased by the FCC. Upon release of the records, FCC defended its decision not to take enforcement action, stating, “given that the RF exposure limit includes a significant safety margin, none of the results suggest there is any RF safety issue with the devices tested” (219). However, the released records also show that a few years prior, the FCC had forced a recall on an Andy 4G Smartphone model due to violations at 170 % of the SAR limit, a far lower exceedance than the 325% reported in the FCC's 2 mm tests (217).

The FCC's lack of transparency regarding these findings had multiple impacts. The FCC did not disclose its 2 mm SAR measurements in its statements filed in two legal cases that centered on the issue, resulting in Telecom wins for both cases (220, 221). The FCC also did not submit its 2 mm data to its then-open inquiry regarding its rules and test procedures, and the agency maintained that changes to its test distances were “unnecessary” (172). However, the 2021 D.C. Circuit decision mandated that the FCC explain how these test procedures remain valid (165), and later that year the agency announced interim guidance with a transition period tightening the minimum distance from 25 mm to 5 mm (222). This change would bring the allowed test distance in line with the European Union, which already moved to 5 mm in 2016 (223, 224). However, as of this drafting of this paper, the guidance has yet to be finalized, and manufacturers can still choose a larger test distance. Further, a 5 mm distance still does not reflect today's real-world body contact usage, and phones that test compliant at 5 mm can still exceed limits in 0 mm body contact positions (213, 225).

### No robust post-market surveillance, measurement, and compliance enforcement

The FCC does not have a robust post-market surveillance, oversight, and enforcement program for compliance with its FCC's regulations. This author's FOIA requests revealed the FCC has performed minimal post-market surveillance to ensure cell phones are compliant with its limits, with only 5 devices SAR tested from 2020 to November 2024 (226). In contrast, France has conducted robust post-market testing since 2012, evaluating hundreds of phones for RFR compliance (213) and triggering recalls or software updates for over 60 devices found to exceed EU limits (227). Some phone models that they tested at body contact reported SAR levels up to three times the EU limit, equivalent to up to 11 times the U.S. FCC's limit (213).

### The case of the Apple iPhone 12

In 2023, France temporarily suspended sales of the iPhone 12 (model A2403) after its surveillance found SAR levels of 5.75 W/kg exceeding EU safety limits when tested in “on-body” scenarios mimicking a phone held in hand or carried in a pants pocket. Soon afterwards, Apple issued a software correction for the model to reduce radiation emissions, but only for the iPhone 12s in France. In 2025, 2 years later, a European Commission ruling then forced Apple to update the models EU-wide (228). The organization Phonegate Alert filed a legal complaint against the French government for failing to promptly inform the EU member states of the findings calling the two-year delay “unacceptable” and requesting corrective measures to be adopted “without delay” (229).

FOIA records released in 2024 document that the FCC had initiated its own iPhone 12 SAR tests when France first suspended sales in 2023, but technical problems delayed the process for months (230). However, the FCC has not released records of the measurements, nor is it known if Apple updated its U.S. models to reduce RFR emissions.

### Cell tower and base station compliance issues

The compliance oversight regulatory gap also extends to wireless infrastructure such as cell towers and 4G/5G small cells, as the US operates on what is essentially the honor system. FCC does not regularly audit base station network antenna emissions, conduct surprise inspections, or review compliance reports. There is no federal requirement for annual or periodic post-installation testing. Wireless providers often submit self-funded engineering simulations, rather than real-world measurements, as proof of compliance. RFR compliance reports lack standardized formats, consistent measurement protocols, and clearly stated enforcement requirements. Many landlords, as well as local and state agencies, assume safety is assured, unaware that there is no oversight to verify compliance or to ensure that recommended compliance measures are implemented.

Exceedances for base station antennas have been documented, especially for building-mounted antennas. As an example, a 2021 measurement study of a rooftop lounge area using professional-grade spectrum management tools documented RFR spikes up to 264% of FCC limits in a Crest Factor analysis (231). A 2014 Wall Street Journal investigation reported on RFR audits that found one in ten rooftop cell sites assessed violated FCC limits (232).

### No EMF measuring or monitoring program

The U.S. no longer has a program to measure and monitor EMF levels, as the last federal report on the issue is an EPA report dated 1986 (233). Independent publications have documented increases since then. A 2018 multi-country study reported RFR levels in Los Angeles at 70 times higher than estimates in the 1985 report (234). A 2021 assessment in Pennsylvania concluded power densities produced by the 5G/4G LTE antennas are 10 to 100 times higher than older networks (235), and hotspots have been documented on the street close to “small cell” antennas in South Carolina (236).

In contrast, several other countries conduct real-time, 24/7 monitoring and/or publish antenna locations/measurements from their audits/spot checks, and many provide the information online on publicly accessible, easy-to-navigate maps. Frances' map presents RFR levels color-coded by intensity and even shows transmitter directions (237). India states it audits up to 10% of sites annually, publicly posting all results, and penalizing providers that exceed limits (238). Greece has a continuous measurement program via 500 sensors located across the mainland and islands, with data posted in an online map (239).

Importantly, such government compliance programs have faced significant criticism regarding rigor and methodology (240). The levels are averaged, omitting peak intensities. Analyses have found that reliance on the posted field measurements and theoretical calculations can underestimate exposure because they often exclude numerous frequency bands (such as from Wi-Fi, GPS, satellites, radar and military applications), and long-term monitoring indicates that the short-term spot measurements are too brief to fully capture the variability of EMF exposure over time (240). Inclement weather, for instance, can boost signal strength, and levels can peak at various times throughout the day. Most importantly, the campaigns generally conclude compliance with ICNIRP-based limits, which is then interpreted as proof of safety, despite the lack of protection for long-term effects (240).

Despite the criticisms, these platforms nonetheless still represent an attempt to measure environmental levels and transparently inform the public, something the U.S. government has failed to do. Regular and continuous environmental monitoring, ensuring assessment of an individual's total dose from all sources, record keeping, and making the data available to the public should be standard practice for non-ionizing EMF as a widespread environmental exposure.

### A lack of public information and consumer awareness

Compounding the U.S. regulatory gaps regarding compliance, enforcement, and monitoring is a striking lack of transparency. Residents often become aware of new wireless infrastructure installations (e.g., 5G small cells and cell towers) only after the facilities have been approved, excluding communities from the decision-making process. While some localities will post locations of wireless facilities online, many do not. There is no federal public registry of all wireless antenna locations along with RFR compliance reports. Thus, the public is not informed of where network antennas are transmitting and what the expected or measured levels are. Public records requests for information are frequently met with delays, denials, or technical documents that the public cannot easily understand. There is also no database of RFR exposure-related complaints submitted to the FCC, nor the FCC's response.

Regarding personal devices, most consumers are unaware that their phones emit RFR and that manufacturer instructions, as detailed in Table 6, state that devices should be used at a specific distance away from the body to comply with FCC limits.

**Table 6:** Manufacturer's instructions for cell phone and wireless devices.

**Brand**	**Model**	**RFR exposure statement**
Apple	16	“iPhone is evaluated in positions that simulate uses against the head, with no separation, and when worn or carried against the torso of the body, with 5 mm separation” (241).
Samsung	Galaxy Z Fold 3 5G	“Body-worn SAR testing has been carried out at a separation distance of 1.5 cm. To meet RF exposure guidelines during body-worn operation, the device should be positioned at least this distance away from the body” (242).
LG	G8	“Body-worn Operation: This device was tested for typical use with the back of the device kept 0.39 inches (1.0 cm) from the body. To comply with FCC RF exposure requirements, a minimum separation distance of 0.39 inches (1.0 cm) must be maintained between the user's body and the back of the device” (243)
Google	Pixel 9 Pro XL	“In the countries where the Specific Absorption Rate (SAR) limit is 1.6 W/kg averaged over one gram of tissue, the highest SAR values for this device type are 0.92 W/kg when used against head with no separation and 0.99 W/kg against body with 1.0 cm (0.4 in) separation…Keep the device away from your body to meet the distance requirement” (244).
Amazon	Echo Smart Speaker	“This device should be installed and operated with a minimum distance of 20 cm between the radiator and your body” (245).
Samsung	HP Printer M301 Series	“While installing and operating this transmitter and antenna combination the radio frequency exposure limit of 1 m W/cm^2^ may be exceeded at distances close to the antenna installed. Therefore, the user must maintain a minimum distance of 20 cm from the antenna at all times. This device cannot be collocated with another transmitter and transmitting antenna (246).”
Verizon	5G internet Gateway router	“To comply with FCC RF exposure compliance requirements, the antenna used for this transmitter must be installed to provide a separation distance of at least 20 cm from all persons (indoor), at least 54 cm from all persons (outdoor), and must not be co-located or operating in conjunction with any other antenna or transmitter (247).”

Local and state efforts to inform the public have been halted. Maine's “The Wireless Information Act,” which required RFR statements on cell phone packaging, initially passed the State House and Senate in 2014, but failed to pass the routine implementation vote after lobbyists convinced lawmakers to switch their votes (248). The San Francisco and Berkeley unanimously passed *Cell Phone Right to Know* ordinances to inform consumers about RFR at the point of sale were halted by industry lawsuits, which argued that such initiatives violated corporate free speech rights (249–251).

Despite numerous requests that the FCC more transparently inform consumers in its 2013–2019 Inquiry, the FCC concluded that existing information “allows users to make informed decisions” (172). The FCC also submitted a statement in the Berkeley legal proceedings stating that the ordinance was federally preempted and “the information provided on its website and in cell phone user manuals is sufficient to inform consumers about the risk of RF exposure, and that additional notices risk “overwarning” and misleading consumers into believing that RF emissions from FCC-certified cell phones are unsafe” (221). As noted earlier, the FCC did not include its findings that SAR limits were exceeded at 2 mm to the Berkeley filing. Without knowledge of RFR, most people routinely carry phones in pockets or against their bodies, unknowingly allowing exposures that could exceed government regulations (213, 214).

### No robust RFR or non-ionizing EMF protection program for workers

The U.S. does not have a coordinated, comprehensive federal program addressing non-ionizing EMF safety in civilian workplaces. OSHA's comments to the FCC stated, “RF emissions are not on OSHA's active regulatory agenda, so we have not conducted a comprehensive literature review or risk assessment on RF hazards” (168).

Decades ago, both the National Institute for Occupational Safety and Health (NIOSH) and the Occupational Safety and Health Administration (OSHA) had active EMF research programs and expertise. In 1995, Robert A. Curtis, then Director of the U.S. Department of Labor OSHA Health Response Team, recommended a Comprehensive RF Protection Program with “…traditional safety and health program elements including training, medical monitoring, protective procedures and engineering controls, signs, hazard assessments, employee involvement, and designated responsibilities for program implementation” (252). It was never implemented.

NIOSH, whose experts previously recommended and developed precautionary measures to minimize EMF risk (253–256), never formally issued policies to employers nationwide nor did it issue a final report regarding its funded projects (2008–2014) to develop cost-effective methods to assess and manage workplace EMF exposure (256). No updates have been issued to its posted online resources, such as its 1996 *EMFs in the Workplace* factsheet (257).

There is a need to fully assess occupational exposures, especially with the evolution of modern technologies. Exposures could be exceeded in occupational settings involving dielectric heating of plastics, security, cosmetics and telecommunications and further research is needed to address 5G, and wireless power transfer (258, 259). Routine compliance checks are not required for building-mounted antennas despite maintenance workers, such as HVAC technicians and window washers, facing heightened exposure when working on rooftops and buildings. While OSHA can cite both employers and landlords, federal and state agencies lack active engagement in educating employers and workers about non-ionizing radiation exposure, leaving many workers unaware of potential risks or how to report them.

Accountability for cell tower climbers, referred to as “the most dangerous job in America,” (260) remains diffuse, fragmented, and often unclear, especially because subcontracting is widespread and infrastructure is being built at a rapid pace. Although OSHA and the FCC have initiated activities to address these issues, preventable accidents continue to occur (261), and RFR is not a part of the conversation. A 2020 case report describes how a telecom engineer who was accidentally overexposed, seven years later developed demyelinating brain lesions and kidney and lung masses, which they believe were a delayed RFR injury, mimicking Multiple Sclerosis (MS) (262).

While the U.S. never implemented a federal program, the IEEE EME Safety Program (C95.7-2022) provides several key elements to assess, manage, and mitigate risk applicable to the workplace (263); however, elements are not required in most U.S. workplaces. In contrast, the European Union issued a 2013 Directive to member states on health and safety requirements regarding EMF exposure to workers, along with an implementation guide (264, 265). Although the Directive does not address long-term effects, it does include risk assessments and preventive measures, with special considerations for pregnant workers and those with medical implants.

Equally important is the need to re-evaluate FCC regulations regarding the separation of human exposure limits into occupational/controlled and general population/uncontrolled exposures. According to the FCC, the “Occupational” limit was designed to allow more exposure because a worker is trained on the issue, aware of their exposure, and able to exercise control over it. The “general public” applies to everyone else, regardless of all ages and health statuses, who are not aware of their exposure, and therefore less likely to control exposures. The assumption that a worker should be allowed to be exposed to higher RFR levels just because they are trained warrants an evidence-based re-evaluation. Since 1996, there has been a substantial increase in wireless infrastructure, impacting a worker's ability, no matter how trained, to control their exposure. Telecommunication technicians/tower climbers report that even though they verify that transmitters are powered down at a facility, they are working on, they can still be exposed to RFR antennas on nearby buildings, and sometimes transmitters are accidentally powered *back on* (266). From a logical standpoint, one could argue that workers should have *more* stringent exposure limits given their more prolonged exposures. Yet without active agency research, such questions are not being investigated in the U.S.

Workers in nearly all of today's modern jobs, such as those in emergency response, education, healthcare, retail, and service sectors, are exposed to chronic low-level RF radiation from wireless devices, including cell phones, headsets, walkie-talkies, Wi-Fi access, computers, boosters, and other wireless equipment. These exposures are not assessed or mitigated, and workers are largely unaware of the exposure.

### No consideration for environmental impacts

U.S. regulations ignore wildlife as they were designed only for humans. Studies have observed that wireless radiation and non-ionizing EMFs can adversely affect wildlife, particularly insects, and impact plant health, yet no U.S. regulations currently exist to safeguard ecosystems from these exposures (185, 267–271). Levitt et al. (2, 185, 186) reports a broad range of impacts and the need to update policy in their three-part review of impacts to flora and fauna, “Numerous studies across all frequencies and taxa indicate that current low-level anthropogenic EMF can have myriad adverse and synergistic effects, including on orientation and migration, food finding, reproduction, mating, nest and den building, territorial maintenance and defense, and on vitality, longevity and survivorship itself. Effects have been observed in mammals such as bats, cervids, cetaceans, and pinnipeds among others, and on birds, insects, amphibians, reptiles, microbes and many species of flora” [(185), abstract].

Compliance test procedures ignore the airspace near antennas where flora and fauna live, e.g., birds and insects fly/nest/perch, or bats fly/feed, and trees grow. RFR levels close to antennas can exceed regulatory limits past 50 feet or far more, but facilities are generally deemed compliant because compliance tests only measure ground-level RFR or where people exist. While experts in wildlife and bioelectromagnetics are calling for safeguards (15, 186, 269, 271) and the European Union is funding research (123, 126), the U.S has never evaluated the impact of wireless densification on flora and fauna.

Additionally, former FCC attorney Erica Rosenberg, who served as Assistant Chief of the Competition and Infrastructure Policy Division at the agency, detailed the way the FCC's approach has systematically failed to fulfill its duties under the National Environmental Policy Act (NEPA) in “multiple and significant ways” (272). She details how the FCC's inadequate procedures have allowed most wireless infrastructure, including cell towers, small cells, and satellite networks, to be categorically excluded from meaningful environmental assessment how the FCC delegates much of the environmental review process to industry itself, with little oversight or tracking, stating the agency “fails to vigorously enforce its rules so that industry noncompliance is rampant” [(272), p. 26].

### Risk mitigation measures and safeguards for the vulnerable

As detailed in the European Environment Agency's *Late Lessons from Early Warnings*, proving harm from corporate pollution can take decades (273), and the cost of waiting can have serious health implications. Roda and Perry considered the growing scientific literature in 2014 and concluded that, despite the incomplete state of scientific knowledge, a precautionary approach regarding base stations near children was aligned with European states' obligations under international human rights law (274). As detailed in Table 4, several countries have implemented policies to mitigate risk to children, regardless of whether health effects were considered proven/established or not. Belgium, like France, banned the sale of cell phones designed for young children, concluding that precaution was needed after the WHO/IARC 2011 classification (275).

Schools are a critical area for risk mitigation measures. France, Israel, Cyprus, and French Polynesia reduce Wi-Fi exposure in young children's classrooms, and numerous countries have banned cell towers on school property (19, 276). Cellular antennas installed near schools can increase the environmental exposures in classrooms (277, 278), and closer proximity to network antennas are associated with cancer, radiofrequency sickness, and altered biochemical markers (22, 279, 280). But while several local U.S. school boards have cell tower bans, there is no U.S. federal policy or guidance. In many economically disadvantaged communities, cell towers are proposed as an ideal way to address financial challenges, and parents have fewer resources to stop such installations.

Numerous international governments also have issued recommendations that parents “should” reduce children's exposure, explicitly stating that children could be vulnerable to effects (19, 179). Although the American Academy of Pediatrics, Santa Clara California Medical Association and Maryland State Council on Children's Environmental Health Protection have issued recommendations for families and schools on how to reduce cell phone radiation exposure (19, 281, 282), U.S. public health agency websites lack clear, straightforward advice and have, in the case of the CDC, even removed mention of the unique vulnerabilities of children (283, 284). The CDC, which previously drafted (284–286) and more recently funded, the development of website content on wireless with industry-tied experts (287, 288), only offers two tips “if you are worried” to reduce RFR exposure (289). The FDA's children and cellphone webpage has an image of a smiling teenager looking at his phone and states, “current scientific evidence does not show a danger to any users of cell phones from radio frequency (RF) energy, including children and teenagers” and offers 4 simple steps “that anyone, including children and teenagers, can take…” omitting information on children's unique vulnerability to environmental exposures (290).

Federal policies must also be strengthened to support people reporting symptoms from exposure (known as electromagnetic hypersensitivity, electromagnetic syndrome or microwave sickness) (3, 187, 280, 291) so that they are properly accommodated under the ADA, and that outdated FCC rules do not override civil rights protections.

### Proposals to address regulatory gaps

In 2012, federal legislation in *The Cell Phone Right To Know Act* HR 6358, was proposed to address several of the above detailed regulatory gaps (292). It would have increased federal research grants for bioeffect research, directed the EPA to develop science-based exposure standards, directed the FDA to promulgate RFR labeling at the point of sale and online, and amended the 1934 Communications Act to exclude a prohibition on state or local government zoning regulation of personal wireless service facilities based on health effects. However, despite strong support by the largest group of children's doctors in the U.S., the American Academy of Pediatrics, the comprehensive legislation failed to make it to a vote (293).

In 2020, a New Hampshire State Commission created by legislation issued a Report on “The Environmental and Health Effects of Evolving 5G Technology” with 15 recommendations summarized in Table 7 to “protect people, wildlife, and the environment from harmful levels of radiation” after a year-long investigation into the subject (113). Numerous scientists provided testimony, and the Commission sent inquiries to federal agencies. The FCC did not respond, and letters from the FDA and NCI revealed their absence of a comprehensive scientific review (294).

**Table 7:** Recommendations of the New Hampshire State Commission 2020 Final Report on Health and Environmental Impacts of 5G and Evolving Technology (113).

**Issue**	**Majority report recommendations**
Scientific research	- Send a resolution to the U.S. Congress to require the FCC to commission an independent health study and review of RFR safety limits. - Establish new measurement protocols to evaluate high data rate, signal characteristics associated with biological effects and summative effects of multiple radiation sources. - Promote basic science and clinical research on electromagnetic hypersensitivity and biological effects, ensuring ADA protections are considered.
Environment protection	- Engage agencies with ecological knowledge to develop RF-radiation safety limits that will protect the trees, plants, birds, insects and pollinators. - Require the FCC, under the National Environmental Policy Act, to do an environmental impact statement as to the effect on New Hampshire and the country from 5G and the expansion of RFR wireless technologies.
Measurements	- Measure RFR intensities systematically throughout the state and develop a publicly accessible map to present the information. - Conduct RFR signal strength measurements for cell sites by independent contractors. - Include RFR measurement training for home inspectors in the Office of Professional Licensure and Certification.
Risk mitigation	- Require setbacks of 1,640 feet for new wireless antennas from residences, businesses and schools. - Equip cell phones and wireless devices with updated software that stops cell phones from radiating when positioned against the body. - Replace Wi-Fi with RFR free internet connections in schools and libraries. - Establish RFR free zones in commercial and public buildings, especially healthcare facilities. - Support statewide deployment of fiber optic cable connectivity with wired connections inside homes.
Awareness and education	- Educate the public on minimizing RFR exposure with multimedia public service announcements on radio, television and print. - Post warning signs in commercial and public buildings. - Label all poles or structures holding 5G antennas in public rights-of-way with RFR warning signs readable from nine feet away.

## Is there evidence of regulatory capture?

There is evidence of a decades-long industry regulatory capture of key U.S agencies, particularly the FCC. As Seymour and Seymour stated in 2013, “This failure to update safety standards in the face of increasing scientific evidence of biological harm to humans and wildlife is a graphic example of the corrupting effect of industry money on Congress, and a perfect example of why huge amounts of industry money compromises democracy” [(159), p. 175].

In 2015, Norm Alster, a journalist on a writing fellowship at the Edmond J. Safra Center for Ethics at Harvard University, published the report *Captured Agency, How the Federal Communications Commission Is Dominated by the Industries it Presumably Regulates*, detailing how the FCC operates under the “undue” influence of the wireless industry, enabled by a revolving door between FCC leadership and the wireless industry lobby and consulting law firms (295). In turn, he says, “the wireless industry has been allowed to grow unchecked and virtually unregulated, with fundamental questions on public health impact routinely ignored” [(295), p. 5]. At that time, Tom Wheeler, former CEO of the Cellular Telecommunications and Industry Association (CTIA), an industry trade group, had become the Chair of the FCC. Like the industry-tied leadership before and after him, Wheeler pursued an aggressive deregulatory agenda, opening up millimeter wave spectrum, stating that American leadership in 5G must be a national priority (296). As Table 8 illustrates, this dynamic remains firmly in place today.

**Table 8:** The revolving door between FCC commissioners and the telecommunication industry examples from 1996 to current.

**Name**	**FCC position**	**Wireless industry tie/position**
Brendan Carr	FCC Chair (2025 to present) FCC Commissioner (2017–2025)	Attorney, Wiley Rein LLP representing telecommunication companies (2005-2007, 2009–2012)
Ajit Pai	FCC Commissioner (2021–2025) FCC Chair (2017–2021) FCC Commissioner (2012–2017)	President/CEO of the CTIA (2025 to present) President, Searchlight Capital Partners which specializes in telecom investments (2021–2025) Verizon Associate General Counsel (2001–2003)
Anna M. Gomez	FCC Commissioner (2023–present)	Partner at Wiley Rein, Media & Technology Practice (2013–2022) Vice President State and Federal Regulatory, Government Affairs, Sprint Nextel 2006–2009
Nathan A. Simington	FCC Commissioner (2020–2025)	Senior Counsel at Brightstar “the #1 specialized wireless distributor in the world” (2017–2022)
Thomas Johnson	FCC General Counsel (2017–2021)	Partner & Co-Chair, Appellate Practice at Wiley Rein LLP (2021–present) Gibson, Dunn & Crutcher (2006–2016)
Bruce Romano	Associate Legal Chief at FCC's Office of Engineering and Technology (OET) (1978–2018 Media Bureau and OET)	Wiley Rein LLP where he assists clients in navigating FCC policies and procedures (302) (2018–at least 2024)
Tom Wheeler	FCC Chair (2013–2017)	President of Shiloh Group LLC, telecom sector investments and strategy (2017–present) President and CEO of the CTIA 1992–2004 President and CEO of the National Cable Television Association (NCTA) (1976–1984)
Meredith Attwell Baker	FCC Commissioner 2009–2011	President and CEO of the CTIA (2014–2015) Director of Congressional Affairs at the CTIA (1998–2000)
Robert M. McDowell	FCC Commissioner (2006–2013)	Partner in Wiley Rein Law firm, Telecom practice (2014–2016) Cooley LLP, now Chair of global communications (2016–present) Senior Vice President and Assistant General Counsel of the Competitive Telecommunications Association (1999–2006) VP & General Counsel America's Carriers Telecommunications Association (ACTA) 1998–1999
Julius Genachowski	FCC Chair (2009–2013) FCC Commissioner (2006–2009)	Partner & Managing Director in global technology, media, and telecom investments at the Carlyle Group (2014–2024) Senior advisor (2024 to present) Board of Directors Sonos Inc (2013–2023, President BOE 2023 to present) Board of Directors Sprint (2015–2020)
Jonathan S. Adelstein	FCC Commissioner (2002–2009)	President & CEO, PCIA, now the Wireless Infrastructure Association (2012–2022)
Kevin J. Martin	FCC Chair (2005–2009) FCC Commissioner (2001–2005)	Interim Head then VP, U.S. Public Policy, Meta/Facebook (2015–present) Partner, Squire Patton Boggs LLP, coleader of Telecom practice group (2009–2015) Partner at Wiley Rein in communications, legislative, and appellate litigation matters (1998–2001)
Kathleen Q. Abernathy	FCC Commissioner (2001–2005)	Board of Directors, DISH Network (2019–present) BAI Communications (2020–2024) Frontier Communication, Board of Directors (2006–2010) then Chief Legal Officer and Executive Vice President (2010–2016)
Michael K. Powell	FCC Chair (2001–2005) FCC Commissioner (1997–2001)	President and CEO of the NCTA (2011–2025 Retirement)
William E. Kennard	FCC Chair (1997–2001) FCC General Counsel (1993–1997)	AT&T Board Lead Independent Director (2025–present) AT&T Board Chair (2021–2025) AT&T Board (starting 2014)

Former FCC Chair Ajit Pai was recently appointed to lead the CTIA, succeeding Meredith Attwell Baker, also a former FCC Commissioner. During his tenure at the FCC, Pai passed sweeping fast-tracking rules that stripped local authority regarding 5G and wireless infrastructure (297). As of this writing, the current FCC Chair is Brendan Carr, an attorney who previously represented telecommunications companies, including the CTIA, in its legal challenge against San Francisco's cell phone right-to-know ordinance (298). Before he was appointed FCC Chair, Carr co-authored a chapter in Heritage Foundation's Project 2025 that included telecommunications industry-friendly recommendations such as further streamlining the infrastructure permitting process, expediting satellite reviews, and limiting government fees (299). Under his leadership, the FCC has already approved measures to accelerate satellite licensing (300), and the agency is poised to act on more wireless streamlining proposals that would weaken environmental review processes and dismantle state and local authority regarding cell towers (301).

To fully understand the close ties between federal regulators and the telecommunications industry, it is critical to also examine the evolution of law firms that consult for the industry. In 1974, President Richard Nixon appointed Richard E. Wiley as FCC Chairman. After leaving the FCC, Wiley co-founded a Washington, D.C. law firm now known as Wiley Rein LLP. The New York Times profiled Wiley in 1992 as a man of significant influence (303) and the Los Angeles Times in 2001 dubbed Wiley the unofficial FCC sixth commissioner (304) due to his impressive impact on telecommunications policy. Wiley Rein LLP, along with the law firm of Gibson, Dunn & Crutcher, represented industry clients, including CTIA—The Wireless Association, in their litigation opposing San Francisco and Berkeley's cell phone right-to-know ordinances (305–307) and they have employed many FCC Commissioners and lawyers as detailed in Table 8.

As an example of the revolving door beyond the Commissioners, Thomas M. Johnson Jr., worked at Gibson Dunn before serving as FCC General Counsel. At the FCC, he was a counsel of record on the FCC's brief against EHT et al. and penned two pivotal FCC statements of interest siding with industry in both the Berkeley cellphone right-to-know case and other litigation centering on consumer awareness of cell phone radiation exposures (220, 221). He then left the FCC to work at Wiley Rein, co-authoring a memo for the CTIA opposing petitions in Massachusetts that would have required routine RFR audits for cell towers, RFR mitigation measures for devices, and educating schoolchildren (308). This revolving-door advocacy illustrates a key strategy detailed in Alster's *Captured Agency* report: the use of aggressive legal tactics by industry to suppress consumer safety initiatives and undermine local regulatory efforts (295).

Studies continue to find that industry-sponsored research is less likely to conclude adverse effects from non-ionizing EMF exposure, while independently funded studies more often report health effects (28, 309–315), mirroring historical patterns seen in other industries such as tobacco, lead, and chemicals (295, 316, 317). This, combined with heavy lobbying, political contributions, and well-funded public relations campaigns, maintains the inaccurate narrative of a scientific consensus on wireless safety (295, 318, 319). Thus, industry can pursue an unrestrained agenda to proliferate wireless networks, unchecked for safety.

## What reforms are needed to address regulatory gaps?

The existing regulatory vacuum can be addressed by common-sense reforms to bring the U.S. regulatory framework into the 21st century so that it reflects current science, modern usage patterns, occupational exposures, and the realities of cumulative exposures from multiple wireless devices and technologies. Robust oversight and enforcement is critical, and updated policies should address both human and ecological health as they are inextricably intertwined. Recommendations to address regulatory deficiencies include:

*Gap: A lack of agency activities investigating human health effects of wireless technology*

*Reform: Reinvigorate national research*.

1. Reinstate a comprehensive national EMF bioeffects research program across key federal agencies with annual reporting.
2. Ensure a thorough ongoing review of the scientific evidence, paired with targeted research activities to address critical data gaps and evaluate industry involvement.
3. Reestablish an interagency group to coordinate among federal agencies.
4. Expand research to encompass all artificial non-ionizing frequencies, including powerlines, charging systems, wireless power transfer, LED systems, satellite systems, and mmWave -THz bands associated with emerging technologies.

*Gap: Human exposure limits are only designed to protect against short-term heating related effects*.

*Reform: Revise limits with an evidence-based approach that protects for effects of non-heating and long-term effects*.

1. Update exposure limits using quantitative risk assessments grounded in current scientific research to address adverse biological effects, including those from cumulative and long-term low-intensity exposures, applying an ecosystem-based approach.
2. Address signal characteristics (such as modulation) and exposure parameters (environment, metal, etc.) beyond frequency and intensity.
3. Apply safety margins sufficiently protective for children, different developmental and medical sensitivities, ensuring safety factors consistent with limit-setting practices used for toxic and carcinogenic exposures.
4. Conduct periodic, independent scientific reviews of regulations through a transparent science-based process led by public health and environmental agencies.

*Gap: No data gathering on exposures or effects*.

*Reform: Establish national surveillance programs*.

1. Measure and monitor EMF levels, prioritizing sensitive areas like schools and conservation areas, with publicly accessible data dashboard.
2. Establish a comprehensive post-market medical, health, and environmental surveillance and reporting program for consumer devices (phones, computers, etc.) and infrastructure (cell towers, 5G, etc.).

*Gap: Premarket and post-market compliance processes lack oversight and do not reflect real-world usage patterns, conditions, or users*.

*Reform: Modernize oversight and compliance*

1. Require rigorous premarket safety testing for all new wireless technologies, considering human and wildlife impacts.
2. Update cell phone and device compliance testing procedures to reflect real-world exposure, including direct contact use, multiple devices/networks functioning at once, and account for various environmental conditions.
3. Perform routine audits of emissions from networks and devices with enforcement measures.
4. Update and standardize RF compliance procedures and reporting for network infrastructure and develop oversight procedures to ensure recommended remediation is completed and that methodologies and measurement tools are empirically validated.

*Gap: A lack of transparency regarding infrastructure/device emissions and agency activities*

*Reform: Ensure publicly accessible information*

1. Develop a national registry and database of all existing and proposed wireless infrastructure installations (towers, 4G, 5G, etc.), including permitting and RF compliance data.
2. Launch an easy-to-use online website with mapping tools to host RF measurements and registry information.
3. Ensure RF complaints to the FCC and the agency's response are publicly available.
4. Update the FCC's authorization site platform so that the general public can more easily navigate RF data.

*Gap: A lack of consumer awareness*

*Reform: Inform the public on exposures and measures to reduce exposure*

1. Require RF and EMF exposure labeling on all wireless devices and electronics (including charging cables) along with clear instructions on mitigating exposure at the point of sale and packaging.
2. Provide robust public health agency guidance on minimizing exposure from all emitting devices, including cell phones, baby monitors, speakers, printers etc., as well as how to use safer alternatives such as Ethernet and corded connections rather than Wi-Fi and Bluetooth.

*Gap: No safeguards to mitigate risk to children and vulnerable groups*

*Reform: Implement measures to reduce exposures in sensitive areas*.

1. Prioritize wired networks, rather than wireless.
2. Issue robust guidance to institutions and local governments on reducing RF exposure for infrastructure and land use planning, as well as in building networks and devices.
3. Prohibit wireless infrastructure on or near schools, daycares, and critical wildlife habitats.
4. Mitigate day care and school exposures (e.g., replacing Wi-Fi with Ethernet and cell phones powered off)
5. Ensure accommodations are implemented in workplaces, educational institutions, public spaces, and residential areas as recommended by medical providers.

*Gap: No oversight or research focused on occupational exposures*.

*Reform: Safeguard workers with a robust occupational RF/EMF protection program*.

1. Establish a comprehensive occupational RF & EMF protection program.
2. Evaluate exposures in broad range of exposed occupations, from telecom, plastic industry, welding, utilities, healthcare, military, to retail and education.
3. Implement medical surveillance, cumulative exposure tracking, and reporting channels.
4. Provide robust employer/worker training.
5. Develop workplace-specific protocols regarding on-site measurements, cumulative exposure tracking, and required risk mitigation with enforcement mechanisms.
6. Include medical and pregnancy-specific protections such as modified duties, exposure minimization, and clear risk communication.
7. Ensure accommodations for the medically vulnerable and persons with implants.

*Gap: A lack of safer technology consumer options and research*

*Reform: Support research, innovation, and safer alternatives*.

1. Fund research on no/low EMF technology design.
2. Provide economic incentives for industry.
3. Ensure safer options are available to consumers. For example, require a plug-and-play option to connect devices with cables and Ethernet instead of wireless.
4. Require manufacturers and developers to show they have researched and integrated EMF reduction strategies in technologies brought to market and network deployment.
5. Consider a federal certificate program for no/low EMF technology and spaces.

*Gap: Limits were not designed for flora or fauna*

*Reform: Develop a regulatory framework to protect wildlife and habitat*.

1. Establish science-based, species-specific exposure limits to protect wildlife from chronic, low-level non-ionizing EMF.
2. Update compliance procedures to include flora and fauna exposure.
3. Update NEPA procedures with processes that ensure thorough evaluation of cumulative RF effects on ecosystems including vulnerable species like pollinators.
4. Designate low-to-no-EMF zones and setback requirements near conservation areas, migratory corridors, wetlands, and ecologically sensitive areas.
5. Measure and monitor EMF in parks, forests, and other ecologically sensitive zones.

*This is a brief summary, as a more comprehensive review and recommendations on protecting wildlife is discussed in a related paper in this Frontiers on Public Health Special Edition, co-authored by TS* (320).

## Discussion and conclusion

What has emerged from this review is a profound failure of governance, with the U.S. as an exemplar of regulatory gaps. While wireless technologies are rapidly advancing to 5G and beyond, U.S. regulatory oversight has failed to keep pace. The current regulatory framework governing wireless technology in the U.S. is outdated, fragmented, and heavily influenced by industry. Assumptions that federal safety limits are current and science-based are inaccurate, as today's guidelines are based on decades-old research, obsolete/incomplete test methods, and a landscape marked by a near-total absence of civilian research, oversight, and enforcement activity (10). Given the ubiquity of wireless in modern workplaces, the lack of a comprehensive occupational RFR/EMF program, exposure research, and medical surveillance represents a serious gap. This issue should command newfound attention.

The existing research paralysis has led to significant regulatory deflection and abdication. Local and state officials defer to federal agencies. Federal agencies defer to one another. Yet, there has not been an evaluation of *all* the scientific evidence regarding the health effects of wireless technologies despite major technological changes and an ever-growing body of science. Agencies that policymakers expect to have studied the issue simply have not done so.

The result is that U.S. regulations exist without an up-to-date review. Exposure limits are based only on protecting short-term exposures, ignoring the realities of today's long-term cumulative exposure and complex modulations and signaling characteristics reported as important variables. Nothing has changed since 2002, when the EPA wrote that “…federal health and safety agencies have not yet developed policies concerning possible risk from long-term, non-thermal exposures” (321).

To rectify the current situation, government oversight must balance industry power. A strong regulatory framework must be built that rests on transparency and robust evidence-based evaluation, free of industry influence. Prevention is the cornerstone of public health, and the U.S. needs to move toward a risk mitigation approach.

Advancing regulatory reforms is not just a matter of good governance but an ethical imperative. The consequences of ignoring the growing science on non-thermal impacts could be severe, not only in for irreversible health impacts, but also for economic impacts, worker productivity, educational outcomes, and environmental damage. The U.S. should take a leadership role in technology safety by putting children, vulnerable groups, and environmental protection at the center of our decision-making process.
