# Supplementary material for: U.S. policy on wireless technologies and public health protection: regulatory gaps and proposed reforms
Source: Front Public Health. 2025 Dec 19;13:1677583. doi: 10.3389/fpubh.2025.1677583 (PMC12758153; doi:10.3389/fpubh.2025.1677583)
Supplement: Supplementary file 1 [file Data_Sheet_1.pdf]

U.S. Policy on Wireless Technologies and Public Health Protection: Regulatory Gaps and Proposed Reforms

Supplement 1

By Theodora Scarato
Environmental Health Sciences
Theodora@ehsciences.org Theodora.Scarato@proton.me

Supplement 1
Table 1: Documentation of U.S. Agency EMF Health Effects Review and Research Activities and Table 2: Summary of 1995 OSHA Presentation on Proposed Elements of a Comprehensive RFR Protection Program

Table 1 Supplement: Documentation of U.S. Agency Non-ionizing EMF Health Effects Review and Research Activities.

This table lists details the near absence of current EMF bioeffect research activities by key U.S. agencies and entities including the Environmental Protection Agency (EPA), National Institute for Occupational Safety and Health (NIOSH), Department of Labor Occupational Safety and Health Administration (OSHA), National Cancer Institute (NCI), Centers for Disease Control and Prevention (CDC), Food and Drug Administration (FDA) and the U.S. Fish and Wildlife Service (USFWS), the U.S. Radiofrequency Interagency Work Group (RFIAWG), FDA's Technical Electronic Product Radiation Safety Standards Committee (TEPRSSC) as well as the recent directive that Health and Human Services (HHS) report on knowledge gaps regarding children's health and electromagnetic fields.

**Documentation of U.S. Agency Wireless and Non-ionizing EMF Health Effects Review and Research Activities.**		
**Agency**	**Review**<br>**Scientific Evaluation of Health Risks or on the Adequacy of U.S. FCC Limits**<br>***Reports Issued Since 1996***	**Summary of Key Recent Bio-effect Activities***
EPA	None since 1984. No ongoing research.	The EPA was repeatedly tasked to develop safety limits for human exposure RFR and non-ionizing EMF and was poised to release recommendations but was ultimately defunded (1–3).<br><br>Years ago, when the FCC first requested comments on RF guidelines, the EPA commented to the FCC that it recommended updating National

		Council on Radiation Protection (NCRP) 1986 report, which reviewed no studies published passed 1983, to incorporate current scientific knowledge on health effects in exposure limit development (4).<br><br>In the mid-nineties the EPA drafted a research review and additionally funded the NCRP in updating its 1996 review. However, both reviews were never released amidst years of controversy (3,5).
CDC	No scientific reviews or risk evaluations. No research activities Evidence of industry involvement.	In 2014, the CDC website page on cell phones briefly recommended precaution but then retracted recommendation and reference to children's risk was deleted (6,7). The CDC page now states "Some organizations recommend caution in cell phone use. More research is needed before we know if using cell phones causes health effects (8)."<br><br>Authors FOIA requests (9,10) reveal an industry consultant was hired to draft public facing information on wireless safety for the CDC website at that time.<br><br>In 2021, CDC awarded a grant to NCRP to draft wireless health webpages (11–13), with the project proposal stating it would be managed by NCRP researchers which have a long history of industry ties (6, 11-13).
NCI	No reports, formal research reviews, risk or hazard evaluations.	While the NCI website has several website pages (14,15) with information on selected studies compiled by an unknown process, most conclude no effects. However, when asked if the website page content constitutes a safety review, the NCI has repeatedly stated, "neither the literature reviews, nor the fact sheets, make safety determinations" (16).<br><br>When asked for the NCI opinion on wireless safety by the New Hampshire State 5G Commission, NCI deferred to other agencies stating that" the FDA and FCC are the responsible federal agencies with authority to issue opinions on the safety of these exposures" and "the NCI is not involved in the regulation of radiofrequency telecommunications infrastructure and devices, nor do we make recommendations for policies related to this technology" (17).
FDA	Only a literature review on cell phones and cancer.<br><br>No formal hazard or risk assessment	Despite FDA website pages on "scientific evidence for cell phone safety" which states that the FDA "regularly analyze[s] scientific studies" (19, 20), the FDA has not released any quantitative risk assessment of all health risks or evaluation of FCC regulations.<br><br>The FDA's only report is a literature review of studies published to 2018 (18).<br><br>The FDAs 2018 report did not include the researchers who were involved. There were no names and affiliations listed and nor were any review panel researchers listed. When this author requested researchers involved in the FDA report, the FDA stated, "there is no authorship provided as the white paper is simply a summary of a literature review" (21).<br><br>The FDA literature review was limited to research on cell phones and cancer and included no evaluation of Wi-Fi, 5G nor the effects of daily ambient exposure from cell towers (18). The agency has not released a literature review or quantitative risk assessment of the current data on cancer, synergistic effects or impacts to the reproductive, immune, cardiac

		and neurological systems, nor shown evaluation of all the science on children's proportionally higher exposures and developmental vulnerabilities.<br><br>While the FDA website pages seems to offer cell tower and 5G safety assurances, FDA staff has stated they have conducted no review of cell tower health effects (22).<br><br>They also have argued that cell tower radiation is not an issue under their jurisdiction stating, "We don't have jurisdiction over cellphone towers since those are environmental emitters" (23).<br><br>In 2000 the FDA entered into a Cooperative Research and Development Agreement (CRADA) with the Cellular Telecommunications & Internet Association (CTIA) which the FDA states resulted in research projects focused on mechanistic studies related to genotoxicity and exposure assessment studies (24,25). The FDA states no association was found between RFR exposure and adverse health effects (24).<br><br>The FDA also contracted with the National Research Council National Academy of Sciences which released a 2008 report on gaps in the scientific evidence with strong recommendations for studies on health effects (cancer, EEG changes, cognitive effects, brain development, immune system, synergistic and cellular and mechanistic) and exposure characterization, especially for children and the developing fetus (26).
NTP	Toxicology studies concluded "clear evidence of cancer" but follow up studies ceased in 2024.	NTP conducted two-year large scale $30 million toxicology studies in rats and mice that reported increased cancers. The research program found significantly increased gliomas and glial cell hyperplasias in the brain and schwannomas and increased Schwann cell hyperplasias in the heart of male rats. Additionally, DNA damage, reduced pup birth weights, and the induction of cardiomyopathy of the right ventricle of the heart were observed (27).<br><br>The NTP website page (54) states that:<br><br>"The NTP studies found that chronic exposure to RFR used by cell phones was associated with:<br>• **Clear evidence of tumors in the hearts of male rats.** The tumors were malignant schwannomas.<br>• **Some evidence of tumors in the brains of male rats.** The tumors were malignant gliomas.<br>• **Some evidence of tumors in the adrenal glands of male rats.** The tumors were benign, malignant, or complex combined pheochromocytoma.<br>• **DNA damage:** Specifically, they found RFR exposure was linked with significant increases in DNA damage in:<br>◦ the frontal cortex of the brain in male mice,<br>◦ the blood cells of female mice, and<br>◦ the hippocampus of male rats."<br><br>NTP stated in 2018 they were initiating follow-up short-term studies to further investigate the DNA damage they observed in the chronic exposure studies. They began constructing test chambers for experiments to identify biomarkers of damage from RFR (28).

		By 2024 NTP stated that all follow-up studies had ceased because it was technically challenging and more resource-intensive than expected. No additional RFR studies are planned" (29).<br><br>Despite the $5.3 million dollar price tag, the exposure system was "disassembled following completion of the studies, and the chambers are not available for other scientists" (30).<br><br>The NTP concluded its press release on the follow up pilot study by stating, 'high-quality studies to understand the effects of RFR exposure on biological responses are needed given the widespread human exposure to RFR associated with cell phone use" (33).
NIOSH	None	The agency has no current EMF bioeffects research activities. It funded projects between 2008- to-2014 to develop cost-effective methods to assess and manage workplace EMF exposure, aiming to reduce potential health risks (33) but never issued a final report online.<br><br>No updates have been issued to the 1996 factsheet *EMFs in the Workplace* nor to its 1998 *Manual for Measuring Occupational Electric and Magnetic Field Exposures* (34,35).
DOL<br>OSHA	None.	1995: OSHA staff recommended a comprehensive RFR protection program that was never implemented (37).<br><br>The agency does not have a dedicated oversight program to enforce FCC standards or mitigate exposures (36,37) and wrote the FCC in response to its RFR inquiry that, "RF emissions are not on OSHA's active regulatory agenda, so we have not conducted a comprehensive literature review or risk assessment on RF hazards" (38).<br><br>In 2003, NIOSH posted a Safety Checklist Program for Schools that asks if RFR sources have been identified and recommends levels be professionally measured, yet most schools have no such assessments in place today (39).
USFWS	No formal risk assessment. Recommended scientific review.	2002: The U.S. Fish and Wildlife Service (USFWS) developed a peer-reviewed research protocol for studying both collision and radiation impacts of communication towers on migratory birds within U.S. national forests (40).<br><br>2007: USFWS biologist Dr. Albert Manville presented to Congress on potential impacts of cell tower RFR radiation on migratory birds and wildlife, calling for targeted U.S. research stating, "USFWS [is] growing concerned about potential impacts of tower radiation on resident and migrating birds and bats, listed species under our jurisdiction, and other potentially impacted living resources including bees" (41).
DOI	No formal review conducted, Requested robust assessment	2014: The Department of Interior (DOI), based on input from USFWS, sent a letter to the National Telecommunications and Information Administration (NTIA) requesting an assessment of impacts of telecommunications infrastructure on migratory birds including from RFR

		radiation impacts in relation to NEPA implementing procedures of the First Responder Network Authority (FirstNet) wireless network (42).<br><br>The technical attachment (42) documented several laboratory studies reporting RFR impacts to birds and stated, "…the electromagnetic radiation standards used by the FCC continue to be based on thermal heating, a criterion now nearly 30 years out of date and inapplicable today." The requested study was never initiated.
FCC	None.	The FCC cannot itself conduct a scientific review as the agency lacks scientific expertise and stated, in its 2013 Inquiry on wireless RFR guidelines, "…since the Commission is not a health and safety agency, we defer to other organizations and agencies with respect to interpreting the biological research necessary to determine what levels are safe" (43).<br><br>The two primary agencies that advise FCC are FDA, which only controls for near-field devices (cell phones and handheld devices), and EPA, which should advise on ambient environmental exposures (like cell towers) but was defunded for nonionizing radiation research in 1995. There is no agency to advise the FCC on health or environmental risks from cell tower far-field exposures.<br><br>FCC's human exposure limits were guidelines adopted in 1996 and remain unchanged. The FCC has also not significantly updated OET Bulletin 65, its 1997 guidance on RF exposure compliance (44). It remains the official FCC compliance document.<br><br>FCC states on its RF Safety webpage (45) that "The FCC does not have the resources or the personnel to routinely monitor the exposure levels due at all of the thousands of transmitters that are subject to FCC jurisdiction. … In addition, the FCC does not routinely perform RF exposure investigations unless there is a reasonable expectation that the FCC exposure limits may be exceeded."<br><br>The FCC does not have a comprehensive, transmitter-specific database for all of the services it regulates."<br><br>2019: FCC's 2019 decision to maintain its human exposure limits was deemed by the DC Circuit as "arbitrary and capricious" in that it did not address evidence submitted to the FCC indicating "non-cancer" effects, children's vulnerability, environmental impacts, and impacts from long-term exposure (46,47).<br><br>While the FCC had argued that there was no need to update its limits (48), the Court found numerous issues raised by the petitioners had been ignored. The Court ordered the FCC to provide a reasoned determination as to whether the submitted record evidence sent to the agency (consisting of over 1000 comments including hundreds of scientific research papers reporting harmful effects) justifies maintaining the 1996 wireless radiation limits, specifically directing the FCC to:<br><br>1. Explain its decision to retain outdated testing procedures for determining compliance of cell phones and wireless devices.<br>2. Address the impacts of RF radiation on:<br>a. Children's health and development,

		b. Long-term exposure effects,<br>c. The ubiquity of wireless devices, and<br>d. New technological developments since 1996<br>e. The environmental impacts
RFIAWG	No report. However, they sent two letters highlighting issues with the limits.	The federal Radiofrequency Interagency Work Group (RFIAWG) was established in 1995 with experts from the FDA, FCC, NIOSH, OSHA, EPA, DoE, NTIA, NIEHS, CDC, and HHS.<br><br>Although the FCC stated to the DC Circuit in their 2021 brief (2020) and 2021 oral argument in EHT et al. v. the FCC (1) the FCC (46, 48) that the RFIWG workgroup has activities ongoing, the EPA stated in 2020 that, “the RFIAWG is an informal forum for exchange of information and the group does not meet to set, or advise on, policy, rulemaking or guidance. The group has not met in more than two years” (49).<br><br>In 1999 and 2003, members of the RFIWG issued a critique of federal limits as “outdated and insufficiently protective,” highlighting 17 critical issues including the lack of biological basis, inadequate dosimetric modeling, failure to address modulated exposures, flaws in the two-tier occupational/public system, insufficient attention to long-term health effects and problems with how averaging over time and tissue volume can mask peak exposures.<br><br>RFIAWG called for a comprehensive scientific review and stricter, science-based standards to better protect public and worker health (50,51).
TEPRSSC	No.	The Technical Electronic Product Radiation Safety Standards Committee (TEPRSSC) Advisory group is supposed to advise the FDA regarding proposed performance standards for electronic products which emit radiation. However, it has not evaluated the science on bioeffects, reviewed the adequacy of FCC limits nor proposed radiation standards.<br><br>The group has been inactive since 2016 with 11 vacancies as of May 2025 (52).
HHS	No.<br><br>However, a 2025 proposal for a report on data gaps is pending.	In 2025, the Report “Make Our Children Healthy Again Strategy Report” was issued by the Make America Healthy Again Commission, established by Executive Order 14212 states regarding Electromagnetic Radiation that:<br><br>“HHS, in partnership with other departments and Federal agencies, will undertake a study on electromagnetic radiation and health research to identify gaps in knowledge, including on new technologies, to ensure safety and efficacy” (53. p. 7)<br><br>However, there is no completed report, and the timeline, scope, involved federal agencies and details have yet to be released.

*Full details of the involvement of various agencies over decades can be found at Microwave News https://microwavenews.com/

Table 2: Summary of 1995 OSHA Presentation on Proposed Elements of a Comprehensive RF Protection Program by Robert A. Curtis, Director US DOL/OSHA Health Response Team, from his presentation on April 12, 1995, at the National Association of Broadcasters Broadcast Engineering Conference, Las Vegas, NV (35).

**Summary of Robert Curtis' Director US DOL/OSHA Health Response Team Proposed Comprehensive RF Protection Program**
1. Utilization of RF source equipment which meet applicable RF and other safety standards when new and during the time of use, including after any modifications.<br>2. RF hazard identification and periodic surveillance by a competent person who can effectively assess RF exposures.<br>3. Identification and Control of RF Hazard Areas.<br>4. Implementation of controls to reduce RF exposures to levels in compliance with applicable guidelines (e.g., ANSI, ICNIRP), including the establishment of safe work practice procedures.<br>5. RF safety and health training to ensure that all employees understand the RF hazards to which they may be exposed and the means by which the hazards are controlled.<br>6. Employee involvement in the structure and operation of the program and in decisions that affect their safety and health, to make full use of their insight and to encourage their understanding and commitment to the safe work practices established.<br>7. Implementation of an appropriate medical surveillance program.<br>8. Periodic (e.g., annual) reviews of the effectiveness of the program so that deficiencies can be identified and resolved.<br>9. Assignment of responsibilities, including the necessary authority and resources to implement and enforce all aspects of the RF protection program.

References

1. Cleveland RF. Memorandum with the Subject: ET Docket 93-62 Ex Parte Presentation by U.S. Environmental Protection Agency. (1995) https://ehsciences.org/wp-content/uploads/2025/05/1995-Briefing-for-the-FCC-by-the-EPA-on-the-Development-of-RF-Exposure-Guidelines.pdf [Accessed July 25, 2025]

2. Trovata ER. Environmental Protection Agency (EPA) Development of Guidelines for Limiting Public Exposure to Radiofrequency (RF) Radiation. (1995) https://ehsciences.org/wp-content/uploads/2025/05/Letter-from-E.-Ramona-Trovata-EPA-Office-of-Radiation-and-Indoor-Air-to-Richard-M.-Smith-Chief-FCC-Office-of-Engineering-and-Technology-June-19-1995-.pdf [Accessed July 7, 2025]

3. Microwave News. EPA To Assess Health Impacts of Weak, Modulated RF/MW Radiation. *Microwave News* (1994)1,11-12. https://microwavenews.com/news/backissues/s-o94issue.pdf

4. Oge MT. EPA Comments to FCC NPRM: Guidelines for Evaluating the Environmental Effects of Radiofrequency Radiation ET. Docket No. 93-62. https://ehsciences.org/wp-

content/uploads/2025/05/EPA-Comments-to-FCC-NPRM-Guidelines-for-Evaluating-the-Environmental-Effects-of-Radiofrequency-Radiation-ET-Docket-No.-93-62.pdf [Accessed May 19, 2025]

5. Draft NCRP Report Seeks Strong Action To Curb EMFs: Committee Cites 2mG Limit as Goal. *Microwave News* (1995)1,11. https://microwavenews.com/news/backissues/j-a95issue.pdf

6. NCRP Pressured CDC To Remove Cell Phone Safety Advice: You Say "Caution," We Say "Precaution," Let's Call the Whole Thing Off. *Microwave News* (2016) https://microwavenews.com/news-center/caution-vs-precaution [Accessed May 27, 2025]

7. Hakim D. At C.D.C., a Debate Behind Recommendations on Cellphone Risk. *N Y Times* (2016) https://www.nytimes.com/2016/01/02/technology/at-cdc-a-debate-behind-recommendations-on-cellphone-risk.html?_r=0

8. Facts About Cell Phones and Your Health. *CDC* (2024) https://www.cdc.gov/radiation-health/data-research/facts-stats/cell-phones.html [Accessed May 27, 2025]

9. Environmental Health Sciences The CDC Hired an Industry Consultant to Draft Website Information on Wireless Health Risk. (2020) https://ehsciences.org/the-cdc-hired-an-industry-consultant-to-draft-website-information-on-wireless-health-risk/

10. CDC Drafts Released under FOIA by CDC To Theodora Scarato. https://ehsciences.org/wp-content/uploads/2025/05/CDC-FOIA-Wireless-Network-Wbsite-Draft-for-CDC-by-Foster-.pdf

11. SC 8-1: Development of NCRP Informational Webpages to Provide Authoritative Information About the Use of Wireless Technology and Current Evidence on Health Effects - NCRP | Bethesda, MD. *Natl Council Radiat Prot Meas* (2022) https://ncrponline.org/program-areas/sc-8-1/ [Accessed July 28, 2025]

12. National Council on Radiation Protection and Measurements. Application to CDC For Radiation Work Released under FOIA. (2021) https://ehsciences.org/wp-content/uploads/2025/05/CDC-Grant-to-NCRP-Released-to-Scarato-under-FOIA-.pdf

13. Lin JC. Health and safety practices and policies concerning human exposure to RF/microwave radiation. *Front Public Health* (2025) 13: doi: 10.3389/fpubh.2025.1619781

14. Electromagnetic Fields and Cancer. *Natl Cancer Inst* (2022) https://www.cancer.gov/about-cancer/causes-prevention/risk/radiation/electromagnetic-fields-fact-sheet [Accessed May 27, 2025]

15. Cell Phones and Cancer Risk Fact Sheet. *Natl Cancer Inst* (2024) https://www.cancer.gov/about-cancer/causes-prevention/risk/radiation/cell-phones-fact-sheet [Accessed May 27, 2025]

16. National Cancer Institute. Clarification on NCI reviews. https://ehsciences.org/wp-content/uploads/2025/05/National-Cancer-Insitute-Cell-phone-safety-Theodora-Scarato-.pdf

17. National Cancer Institute Letter. New Hampshire State Report on 5G Health and Environment. New Hampshire Commission. https://gc.nh.gov/statstudcomm/committees/1474/reports/5G%20final%20report.pdf

18. Review of Published Literature between 2008 and 2018 of Relevance to Radiofrequency Radiation and Cancer. (2020) https://www.fda.gov/media/135043/download

19. Food and Drug Administration (FDA. Do Cell Phones Pose a Health Hazard. *FDA.gov* https://www.fda.gov/radiation-emitting-products/cell-phones/do-cell-phones-pose-health-hazard

20. Food and Drug Administration (FDA. Scientific Evidence for Cell Phone Safety. *FDA.gov* https://www.fda.gov/radiation-emitting-products/cell-phones/scientific-evidence-cell-phone-safety

21. Theodora Scarato Email Communications with FDA on Authorship of FDA Literature Review Feb 2020 Emails. https://ehsciences.org/wp-content/uploads/2025/11/Theodora-Scarato-Requests-to-FDA-on-Authorship-of-the-FDA-2018-Literature-Review-Feb-to-March-Letters.pdf

22. Email Communications to and from the FDA on Cell Tower Safety. Environmental Health Sciences https://ehsciences.org/wp-content/uploads/2025/06/FCC-FDA-Cell-Tower-Health-and-Safety-Review-Comunication.pdf

23. June 20, 2016 Email from David Kassiday of the FDA From Theodora Scarato FOIA request #: 2017-3549 https://ehsciences.org/wp-content/uploads/2025/08/FDA-FOIA-Daniel-Kassiday-.pdf

24. Cooperative Research and Development Agreement (CRADA). *FDA* (2018) https://www.fda.gov/radiation-emitting-products/cell-phones/cooperative-research-and-development-agreement-crada [Accessed October 9, 2025]

25. United States General Accounting Office. Research and Regulatory Efforts on Mobile Phone Health Issues GAO-01-545. (2001) https://www.gao.gov/assets/gao-01-545.pdf

26. National Research Council. *Identification of Research Needs Relating to Potential Biological or Adverse Health Effects of Wireless Communication*. Washington, D.C.: National Academies Press. (2008). doi: 10.17226/12036

27. Melnick RL. Commentary on the utility of the National Toxicology Program study on cell phone radiofrequency radiation data for assessing human health risks despite unfounded criticisms aimed at minimizing the findings of adverse health effects. *Environ Res* (2019) 168:1–6. doi: 10.1016/j.envres.2018.09.010

28. National Toxicology Program (US). Cell Phone Radio Frequency Studies Factsheet 2023 Way Back Machine. (2025) https://web.archive.org/web/20230308010636/https://www.niehs.nih.gov/health/materials/cell_phone_radiofrequency_radiation_studies_508.pdf [Accessed May 30, 2025]

29. Cell Phone Radio Frequency Studies. Research Triangle Park (NC): National Toxicology Program (US). (2024). https://www.niehs.nih.gov/sites/default/files/NTP_cell_phone_factsheet_jan_2024_508.pdf [Accessed May 30, 2025]

30. Scarato Communications with NIEHS RE NTP follow-up Studies. (2025) https://ehsciences.org/wp-content/uploads/2025/09/Scarato-Communications-with-NIEHS-RE-NTP-follow-up-Studies.pdf

31. Wyde M, Hooth M, Roberts G, Shipkowski K, Shockley K, Smith-Roe S, Stout M, Walker N, Capstick M, Kuster N, et al. Development and Testing of a Novel Whole-body Exposure System for Investigative Studies of Radiofrequency Radiation in Rodents. (2025). https://www.niehs.nih.gov/research/atniehs/dtt/assoc/reports/cellphonerfr

32. Development and Testing of a Novel Whole-body Exposure System for Investigative Studies of Radiofrequency Radiation in Rodents. *Natl Inst Environ Health Sci* https://www.niehs.nih.gov/research/atniehs/dtt/assoc/reports/cellphonerfr [Accessed October 9, 2025]

33.Center for Disease Control C. NIOSH Program Portfolio: NORA Manufacturing Sector Strategic Goals. https://web.archive.org/web/20170628225341/http://www.cdc.gov/niosh/programs/manuf/noragoals/projects/927ZHNK.html

34.Center for Disease Control C. EMFs In The Workplace: DHHS (NIOSH) Publication Number 96-129. (1996). https://www.cdc.gov/niosh/docs/96-129/default.html [Accessed May 30, 2025]

35. National Institute for Occupational Safety and Health**.** Manual for measuring occupational electric and magnetic field exposures. (2023) https://www.cdc.gov/niosh/docs/98-154/default.html

36. Occupational Safety and Health Administration O. Radiofrequency and Microwave Radiation: Overview. https://www.osha.gov/radiofrequency-and-microwave-radiation [Accessed May 25, 2025]

37. Curtis R. Elements of a Comprehensive RF Protection Program: Role of RF Measurements. *Broadcast Eng Conf* (1995) https://www.osha.gov/radiofrequency-and-microwave-radiation/role-of-rf-measurements [Accessed May 25, 2025]

38. William Perry. OSHA comments on the FCC's Notice of Inquiry of March 29, 2013. (2015) https://ehsciences.org/wp-content/uploads/2025/05/Department-of-Labor-to-the-FCC-Letter-on-RF-Radiation.pdf

39. Center for Disease Control C. Nonionizing Radiation: DHHS (NIOSH Publication Number 2004-101). (2003). https://www.cdc.gov/niosh/docs/2004-101/chklists/r1n55r~1.htm [Accessed May 30, 2025]

40. Albert Manville. Protocol for monitoring the impact of cellular telecommunication towers on migratory birds within the Coconino, Prescott, and Kaibab National Forests, Arizona. Research Protocol Prepared for U.S. Forest Service Cellular Telecommunications Study. U.S. Fish and Wildlife Service. (2002).

41. Albert Manville. U.S. Fish & Wildlife Service Concerns Over Potential Radiation Impacts of Cellular Communication Towers on Migratory Birds and Other Wildlife – Research

Opportunities. (2007) https://ehsciences.org/wp-content/uploads/2025/05/Cell-Tower-Wildlife-Effects-US-Fish-and-Wildlife-Service-A.-Manville-Congress-Briefing.pdf

42. Willie Taylor. Department of the Interior Letter and Attachments on FirstNet. (2014) https://www.ntia.doc.gov/files/ntia/us_doi_comments.pdf

43. Federal Communications Commission (FCC). First Report and Order, Further Notice of Proposed Rule Making, and Notice of Inquiry. FCC 13-39. (2013) https://docs.fcc.gov/public/attachments/FCC-13-39A1.pdf

44. Robert F. Cleveland, Jr., David M. Sylvar, Jerry L. Ulcek. OET Bulletin 65: Evaluating Compliance with FCC Guidelines for Human Exposure to Radiofrequency Electromagnetic Fields. (1997) https://transition.fcc.gov/Bureaus/Engineering_Technology/Documents/bulletins/oet65/oet65.pdf

45. RF Safety FAQ | Federal Communications Commission. https://www.fcc.gov/engineering-technology/electromagnetic-compatibility-division/radio-frequency-safety/faq/rf-safety [Accessed October 5, 2025]

46. Court of Appeals for the D.C. Circuit Court. Oral Argument for EHT et al v FCC, docket number 20-1025. (2021) https://www.courtlistener.com/audio/74208/environmental-health-trust-v-fcc/

47. Environmental Health Trust et al. v. Federal Communications Commission. (2021). https://www.fcc.gov/document/dc-circuit-decision-environmental-health-trust-v-fcc [Accessed July 27, 2025] See also a summary at https://ehsciences.org/lawsuit-wireless-radiation-safety/

48. Federal Communications Commission. Brief on Petitions for Review of an Order of the Federal Communications Commission. Environmental Health Trust et al. v. Federal Communications Commission and United States of America. Nos. 20-1025 and 20-1138. (U.S. Court of Appeals for the District Court of D.C. 2020). (2020) https://ehsciences.org/wp-content/uploads/2025/10/FCC-Brief-in-EHT-v-FCC-11-9-2020-1.pdf

49. Lee Ann Veal. EPA answering 12 Questions about the EPA's Information on EMF. (2020) https://ehsciences.org/wp-content/uploads/2025/05/2023-2020-EPA-RF-Radiation-Scarato.pdf

50. Gregory Lotz. Radiofrequency Interagency Work Group (RFIAWG) comments on RF exposure guidelines to Mr. Richard Tell Chair, IEEE SCC28 (SC4). (1999) https://ehsciences.org/wp-content/uploads/2025/05/1999-2003-Radio-frequency-Interagency-Workgroup-Letters-.pdf

51. Norbert Hankin. Radiofrequency Interagency Work Group (RFIAWG) letter with additional issues for the ICES consideration sent to C. K. Chou of Motorola. (2003) https://ehsciences.org/wp-content/uploads/2025/07/2003-Radio-frequency-Interagency-Workgroup-Letter-.pdf

52. Food and Drug Administration (FDA). Past Meeting Materials, Technical Electronic Product Radiation Safety Standards Committee. *FDA.gov* https://www.fda.gov/advisory-committees/technical-electronic-product-radiation-safety-standards-committee/past-meeting-materials-technical-electronic-product-radiation-safety-standards-committee

53. White House. *The MAHA Strategy.* (2025) Available at: https://www.whitehouse.gov/wp-content/uploads/2025/09/The-MAHA-Strategy-WH.pdf (Accessed: 27 July 2025)

54. National Toxicology Program (US). Cell Phone Radio Frequency Studies Website https://ntp.niehs.nih.gov/research/topics/cellphones [Accessed May 30, 2025]
